# Supplementary material for: Accelerating the solar-thermal energy storage via inner-light supplying with optical waveguide
Source: Nat Commun. 2023 Jun 12;14:3456. doi: 10.1038/s41467-023-39190-1 (PMC10261122; doi:10.1038/s41467-023-39190-1)
Supplement: Supplementary file 1 — Supplementary Information [file 41467_2023_39190_MOESM1_ESM.pdf]

Supplementary Information

Accelerating the solar-thermal energy storage via inner-light supplying with optical waveguide

Zhang et al.

Supplementary Movie

The thermal process of the paraffin-graphene composite in inner-light-supply mode during four phase transition cycles got by IR thermal imager.

Supplementary Information

Supplementary Figures, Supplementary Notes and Supplementary References

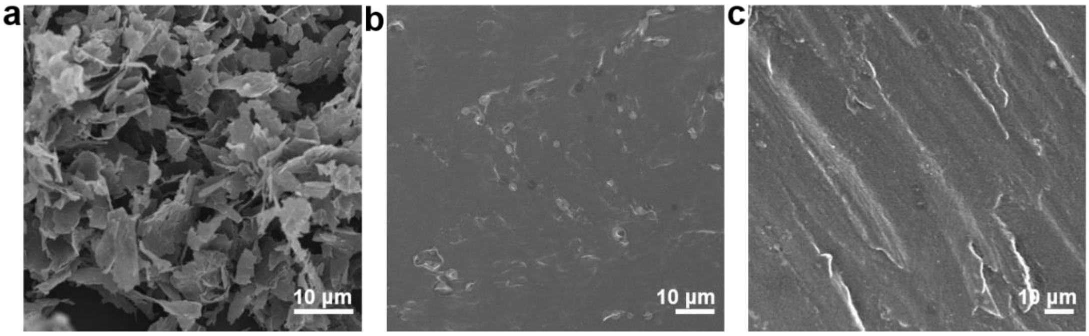

Supplementary Fig. 1 Surface morphology of paraffin-graphene composites. **a-c,** SEM image of **(a)** graphene, **(b)** paraffin and **(c)** the paraffin-graphene composite.

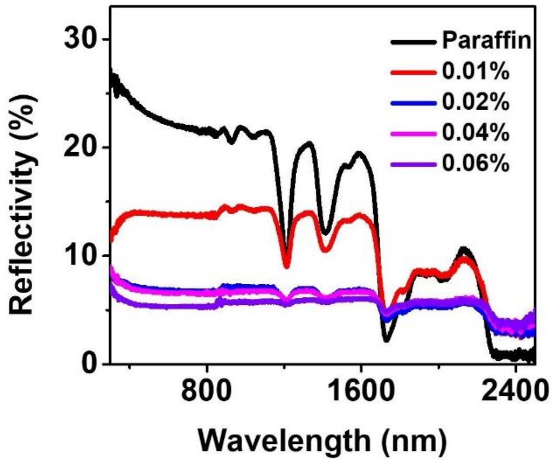

Supplementary Fig. 2 Reflectivity spectra of paraffin-graphene composites with a thickness of 1 mm in the range of 300 to 2500 nm with different loading of graphene.

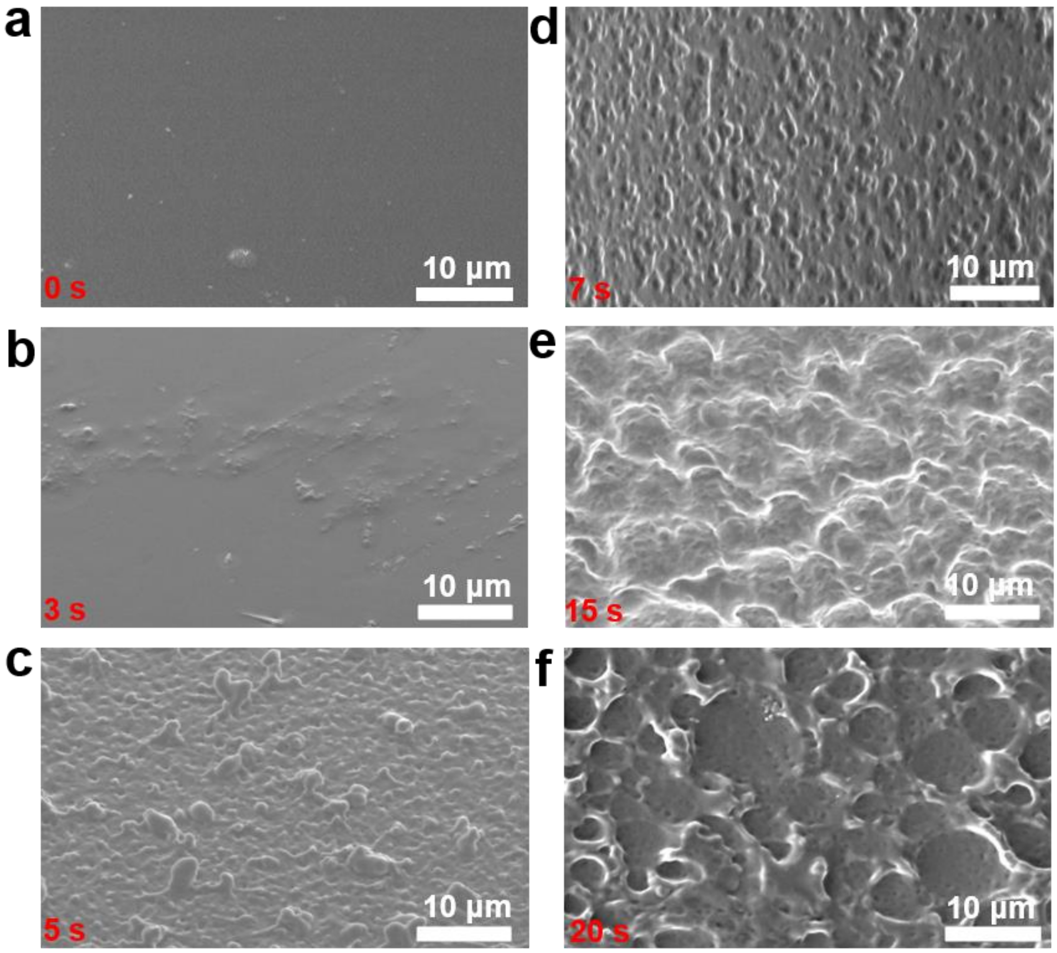

Supplementary Fig. 3 SEM images of PMMA fiber after etching in organic solvents for different period times. **a**, 0 s. **b**, 3s. **c**, 5s. **d**, 7s. **e**, 15s. **f**, 20s.

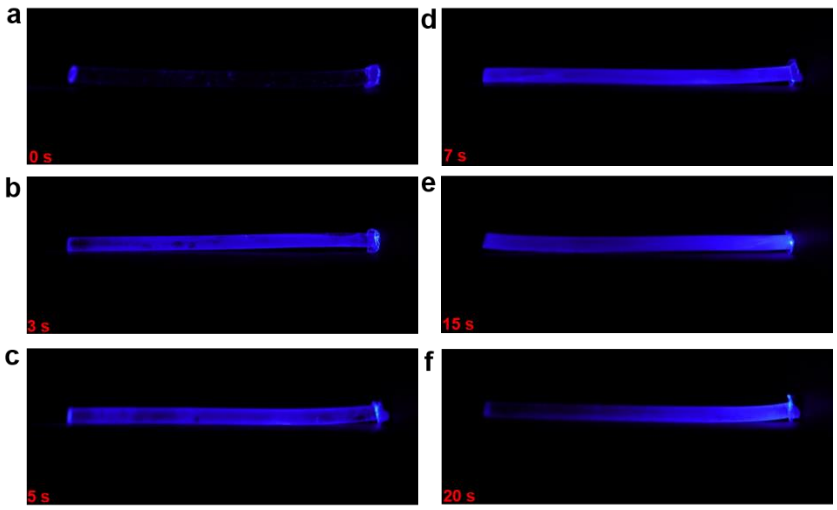

Supplementary Fig. 4 The side-glowing optical photographs of the POF after etching in organic solvents for different period times. **a**, 0 s. **b**, 3s. **c**, 5s. **d**, 7s. **e**, 15s. **f**, 20 s.

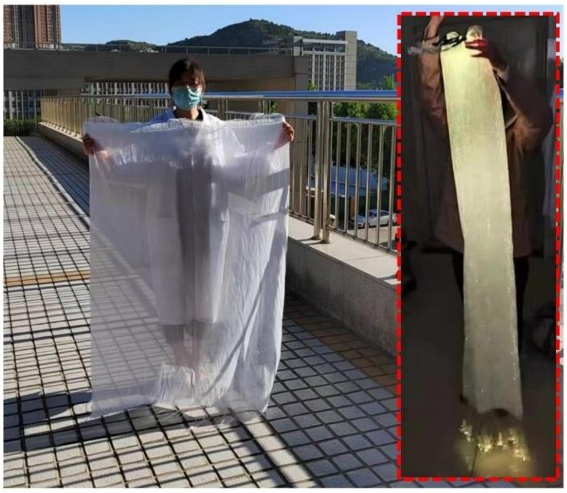

Supplementary Fig. 5 The photograph of large-area commercial optical waveguide fiber cloth.

The inset shows the side-glowing property of the fiber cloth.

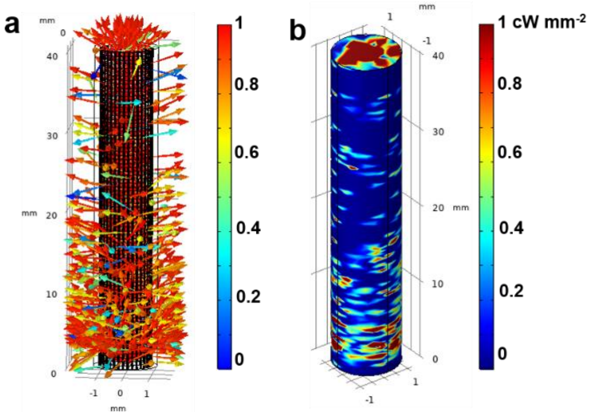

Supplementary Fig. 6 Theoretical modeling of the side glowing property of POF. a, Ray trajectories in POF with pothole of which the diameter is 45 μm on the surface. **b,** Deposited ray power on the side face and bottom surface of POF with pothole of which the diameter is 45 μm on the surface.

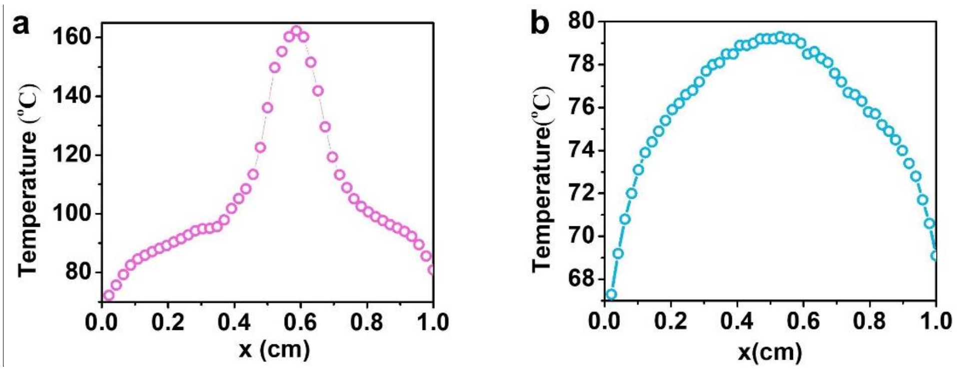

Supplementary Fig. 7 Temperature distribution profiles extracted from the IR thermal images of the top surface of paraffin-graphene composites. a, The temperature distribution along the surface of the quartz cuvette in surface irradiation mode. **b,** The temperature distribution along the surface of the quartz cuvette in inner-light-supply mode.

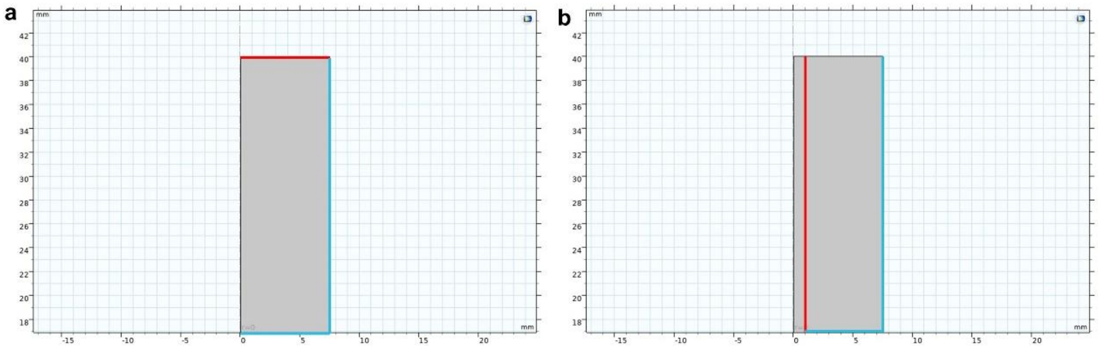

Supplementary Fig. 8 Heat transfer models built in COMSOL. **a, b,** COMSOL heat transfer model geometry for **(a)** surface irradiation mode and **(b)** inner-light-supply mode.

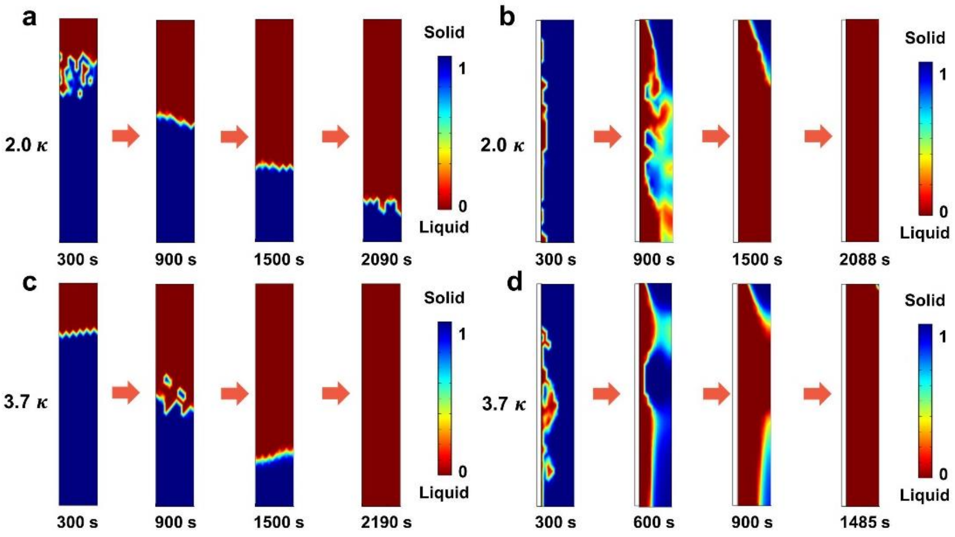

Supplementary Fig. 9 Simulated phase state distribution of paraffin-graphene composites with different thermal conductivity. a, b When the thermal conductivity is set to 2 times of original PCM, the melting process in surface irradiation mode **(a)** and in inner-light-supply mode **(b)**. **c, d** When the thermal conductivity is set to 3.7 times of original PCM, the melting process in surface irradiation mode **(c)** and in inner-light-supply mode **(d)**. Zero stands for liquid state and one stands for solid state in the scale bar.

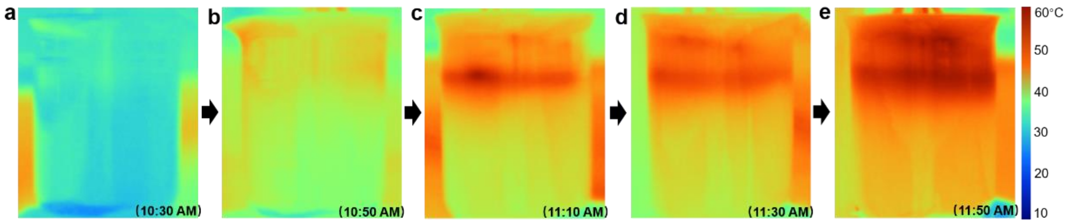

Supplementary Fig. 10 Time-sequential IR images of the large-area paraffin-graphene composites during charging process. **a-e,** IR images of the paraffin-graphene composite after charging for **(a)** 0 min, **(b)** 20 min, **(c)** 40 min, **(d)** 60 min, and **(e)** 80 min.

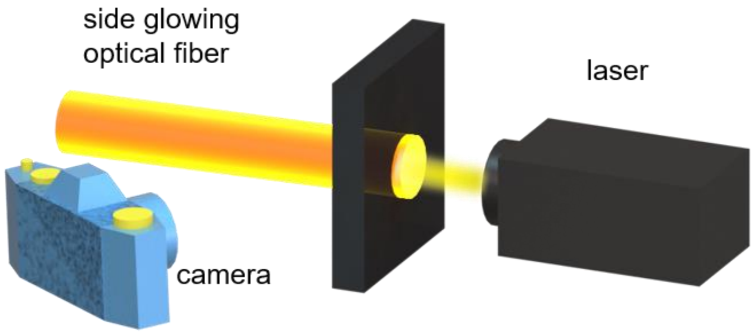

Supplementary Fig. 11 Schematic of experimental setup for testing the side glowing properties of the POF.

Supplementary Note 1: Numerical Simulation

COMSOL Simulation of ray trajectories

The light transmission process in optical fiber obeys the Snell's Law:

$$\frac{n_1}{n_2} = \frac{\sin\theta_2}{\sin\theta_1} \tag{1}$$

where n is the index of refraction, θ_1 and θ_2 is the angel of incidence and refraction. The angel of incidence will be affluence by the pothole at the surface of the optical fiber. The effect of the pothole size on the transmittance is investigated with COMSOL Multiphysics® 5.6 using the Geometrical Optics mode. The transmittance of the pipe is measured by using a Wall node with the Deposited Ray Power subnode to compute the incident power. The optical fiber is composed of PMMA and the refractive index of PMMA was obtained from the experimental data of Sultanova[1]. The diameter and length of the pipe here is 2 mm and 40 mm, respectively. An array of pothole is uniformly distributed at the surface of the pipe, with 36 columns and 400 rows. The rays are released from a point source at one end of the pipe with a core angle of π/6. To save computational time and energy, the size of the pothole in the numerical simulations is larger than that in the actual pipe, and the amount of the pothole in the numerical simulations is less than that in the actual pipe. Fig. S6 shows the ray trajectories and deposited ray power when the diameter of the pothole is 45 μm.

COMSOL Simulation of temperature distribution in PCM

During the solar-thermal energy storage process, solar energy is firstly transferred to thermal energy and the heat transfer in PCM could be described by

$$E_{in} = \rho C_P \frac{\partial T}{\partial t} + \nabla \cdot (k\nabla T) + \varepsilon\sigma \left({T_0}^4 - T_{amb}^4 \right) + h\left(T_0 - T_{amb} \right) \quad (2)$$

where E_{in} represents the solar-thermal energy, ρ is the mass density, C_P is the thermal capacity, T is the local temperature of PCM, k is the thermal conductivity of PCM. The numerical simulations are conducted by COMSOL Multiphysics® 5.6 under the steady and transient analysis mode. The geometric structure of PCM can be simplified as an 2D axial symmetry model as shown in Fig. S8. The latent heat of phase change of the PCM is 151 kJ kg^{-1} and phase-transition temperature is 315.15 K. Under surface irradiation mode and inner-light-supply mode, the PCM samples were surrounded by insulation as green lines shown in Fig. S8 except the top surfaces. The same quantity of heat was input from the top surface under the surface irradiation mode and from the side face under the inner-light-supply mode as red lines shown. For the simulation, the software requires a transition interval between phase 1 (solid phase) and phase 2 (liquid phase). According to the results of DSC tests, the main portion of phase transition takes place over a range of 10 °C (Fig. 2c).

Supplementary Note 2: Thermal charging rate and Solar-thermal conversion efficiency

The charging rate is confirmed by the translational speed of the charging interface. As shown in Figure 4c and 4d, the lines of temperature distribution at different time can be extracted from the infrared photo (Figure 4b). Then a cyan line denoting the phase change temperature of the composite (42°C) is used to tracked the position of the charging interface. The points of where these two lines intersect represent the position

of the charging interface. The translational speed of these points represents the charging rate.

In the solar-thermal storage system, the PCM is surrounded by the thermal insulating polystyrene foam except the top surface. In ideal conditions, the heat dissipation mainly exists at the exposed top surface. Other heat loss, such as increasing the temperature of the container or heating up the insulation foam, is negligible. So the efficiency can be described as:

$$\eta = 1 - \frac{Q_r + Q_c}{E_{in}} \tag{3}$$

$$Q_r = A\varepsilon\sigma\left({T_0}^4 - T_{amb}^4\right) \tag{4}$$

$$Q_c = Ah\left(T_0 - T_{amb}\right) \tag{5}$$

where Q_r and Q_c is radiative and convective heat energy loss from the top surface of container, E_{in} is the solar-thermal energy, A is the surface area of the top surface, T_0 is the surface temperature of PCM, T_{amb} is the ambient temperature, ε is the surface emissivity of PCM (assumed to be 1), σ is the Stefan-Boltzmann constant and h is the heat transfer coefficient (assumed to be 10 W m^{-2} K^{-1}).

In the indoor experiment, the power of incident light is 800 mW and the ambient temperature is 30°C. According to the temperature distribution of the PW-composite samples in Fig. 4b, we extracted the temperature profiles (Supplementary Fig. 7). It can be calculated with the numerical integration algorithm that the convection heat loss in the inner-light-supply mode is 23 mW and that of the surface irradiation mode is 125.4 mW. Moreover, taking the thermal emittance of 1, the radiative heat loss of the composites under these two modes is 18 mW and 147.44 mW, respectively.

Supplementary references

1. Sultanova, N. G., Kasarova, S. N., Nikolov, I. D. Dispersion Properties of Optical Polymers. *Acta Phys. Pol. A* **116**, 585-587 (2009).
