# Supplementary material for: Accelerating the solar-thermal energy storage via inner-light supplying with optical waveguide
Source: Nat Commun. 2023 Jun 12;14:3456. doi: 10.1038/s41467-023-39190-1 (PMC10261122; doi:10.1038/s41467-023-39190-1)
Supplement: Supplementary file 3 — Description of Additional Supplementary Files [file 41467_2023_39190_MOESM3_ESM.pdf]

Description of Additional Supplementary Files

File Name: Supplementary Movie 1
Description: The thermal process of the paraffin-graphene composite in inner-light-supply mode during four phase transition cycles got by IR thermal imager.
