# Supplementary material for: Accelerating the solar-thermal energy storage via inner-light supplying with optical waveguide
Source: Nat Commun. 2023 Jun 12;14:3456. doi: 10.1038/s41467-023-39190-1 (PMC10261122; doi:10.1038/s41467-023-39190-1)
Supplement: Supplementary file 5 — Lasing Reporting Summary [file 41467_2023_39190_MOESM5_ESM.pdf]

nature research

Corresponding author(s): NCOMMS-22-42882B

Lasing Reporting Summary

Nature Research wishes to improve the reproducibility of the work that we publish. This form is intended for publication with all accepted papers reporting claims of lasing and provides structure for consistency and transparency in reporting. Some list items might not apply to an individual manuscript, but all fields must be completed for clarity.

For further information on Nature Research policies, including our data availability policy, see Authors & Referees.

Experimental design

Please check: are the following details reported in the manuscript?

1. Threshold

Plots of device output power versus pump power over a wide range of values indicating a clear threshold
- [ ] Yes
- [x] No

We used commercial laser here, and only cared about the output power.

2. Linewidth narrowing

Plots of spectral power density for the emission at pump powers below, around, and above the lasing threshold, indicating a clear linewidth narrowing at threshold
- [ ] Yes
- [x] No

We only cared about the power density of the commercial laser.

Resolution of the spectrometer used to make spectral measurements
- [ ] Yes
- [x] No

We did not make spectral measurement of the laser.

3. Coherent emission

Measurements of the coherence and/or polarization of the emission
- [ ] Yes
- [x] No

We used commercial laser as light source and only cared about the output power.

4. Beam spatial profile

Image and/or measurement of the spatial shape and profile of the emission, showing a well-defined beam above threshold
- [ ] Yes
- [x] No

We used commercial laser as light source and only cared about the output power.

5. Operating conditions

Description of the laser and pumping conditions
Continuous-wave, pulsed, temperature of operation
- [ ] Yes
- [x] No

We used commercial laser as light source and only cared about the output power.

Threshold values provided as density values (e.g. W cm^{-2} or J cm^{-2}) taking into account the area of the device
- [x] Yes
- [ ] No

In the caption of "Characterization of thermal energy storage. ".

6. Alternative explanations

Reasoning as to why alternative explanations have been ruled out as responsible for the emission characteristics
e.g. amplified spontaneous, directional scattering; modification of fluorescence spectrum by the cavity
- [ ] Yes
- [x] No

We used commercial laser as light source.

7. Theoretical analysis

Theoretical analysis that ensures that the experimental values measured are realistic and reasonable
e.g. laser threshold, linewidth, cavity gain-loss, efficiency
- [ ] Yes
- [x] No

We used commercial laser as light source.

8. Statistics

Number of devices fabricated and tested
- [ ] Yes
- [x] No

We only used commercial laser as light source.

Statistical analysis of the device performance and lifetime (time to failure)
- [ ] Yes
- [x] No

We only used commercial laser as light source.
