# Supplementary figures and images for: Accelerating the solar-thermal energy storage via inner-light supplying with optical waveguide
Source: Nat Commun. 2023 Jun 12;14:3456. doi: 10.1038/s41467-023-39190-1 (PMC10261122; doi:10.1038/s41467-023-39190-1)

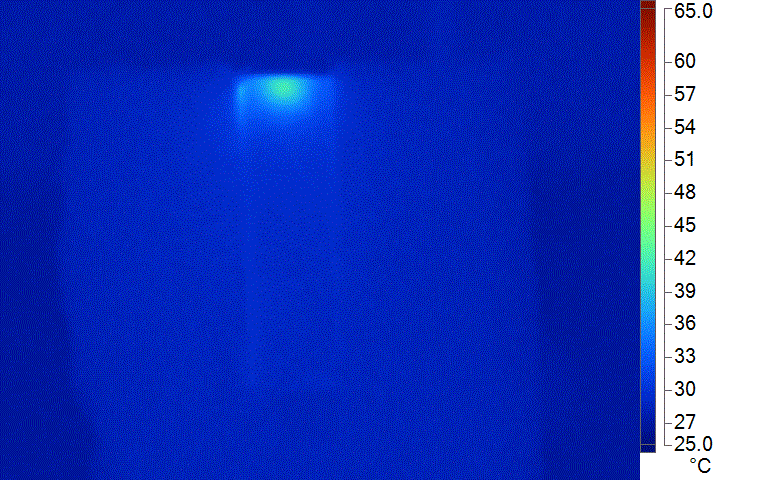

Supplement: Supplementary file 6 — Source Data [file 41467_2023_39190_MOESM6_ESM.zip › 1-original data/fig. 4/fig.4b-up/0s.gif]

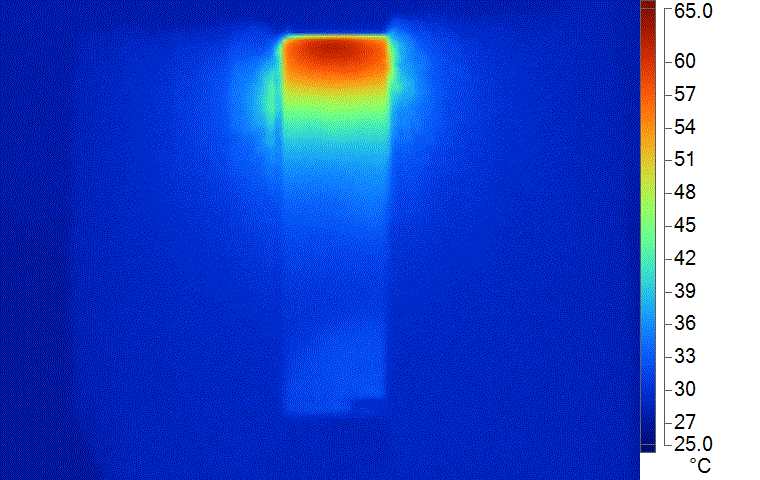

Supplement: Supplementary file 6 — Source Data [file 41467_2023_39190_MOESM6_ESM.zip › 1-original data/fig. 4/fig.4b-up/10s.gif]

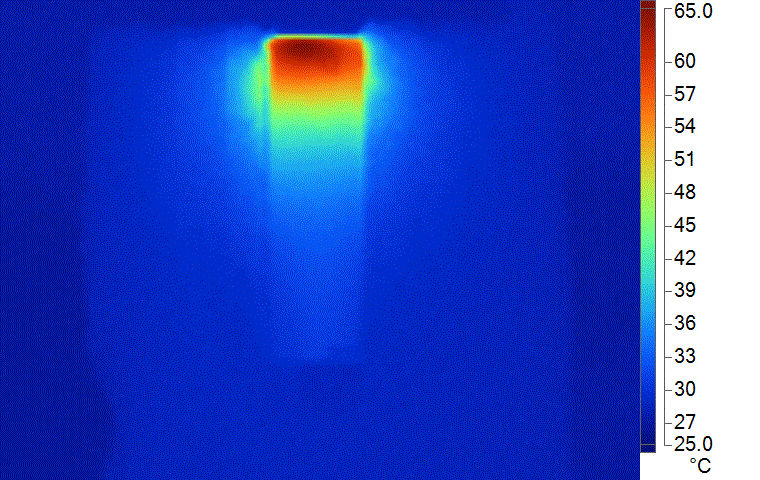

Supplement: Supplementary file 6 — Source Data [file 41467_2023_39190_MOESM6_ESM.zip › 1-original data/fig. 4/fig.4b-up/15s.gif]

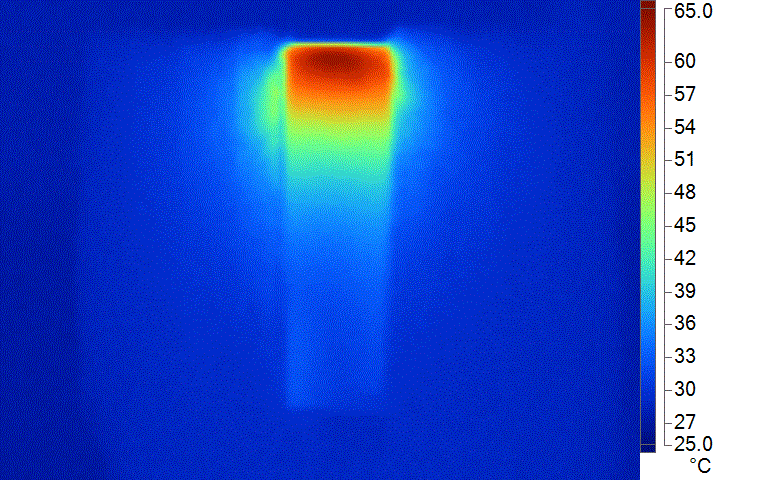

Supplement: Supplementary file 6 — Source Data [file 41467_2023_39190_MOESM6_ESM.zip › 1-original data/fig. 4/fig.4b-up/20s.gif]

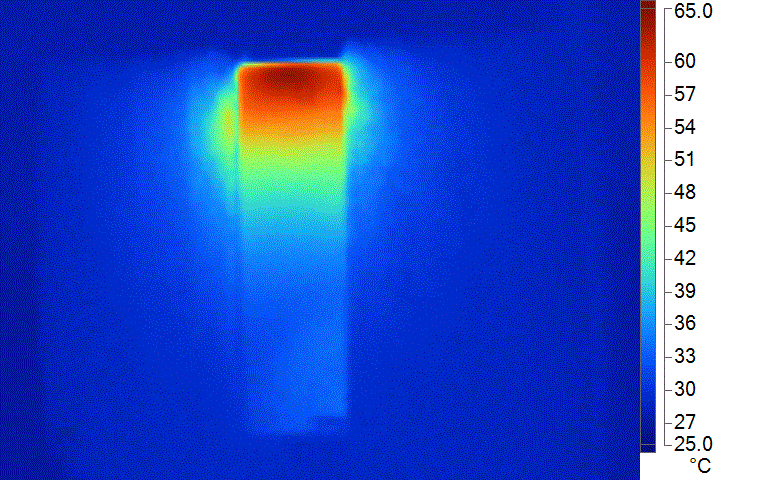

Supplement: Supplementary file 6 — Source Data [file 41467_2023_39190_MOESM6_ESM.zip › 1-original data/fig. 4/fig.4b-up/25s.gif]

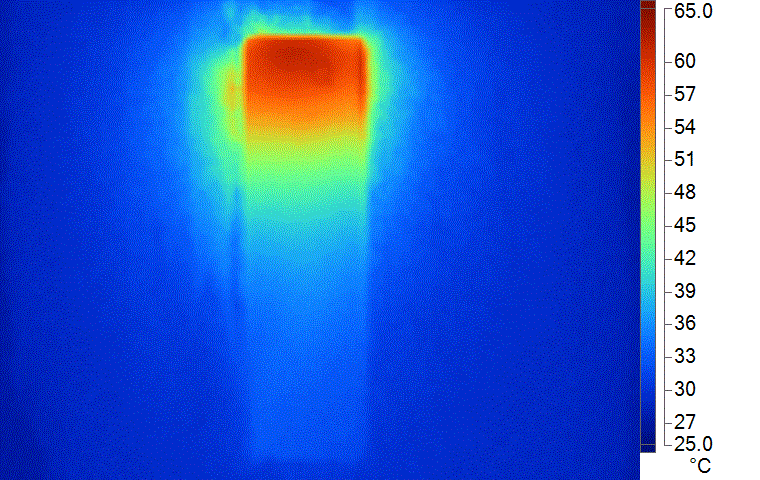

Supplement: Supplementary file 6 — Source Data [file 41467_2023_39190_MOESM6_ESM.zip › 1-original data/fig. 4/fig.4b-up/30s.gif]

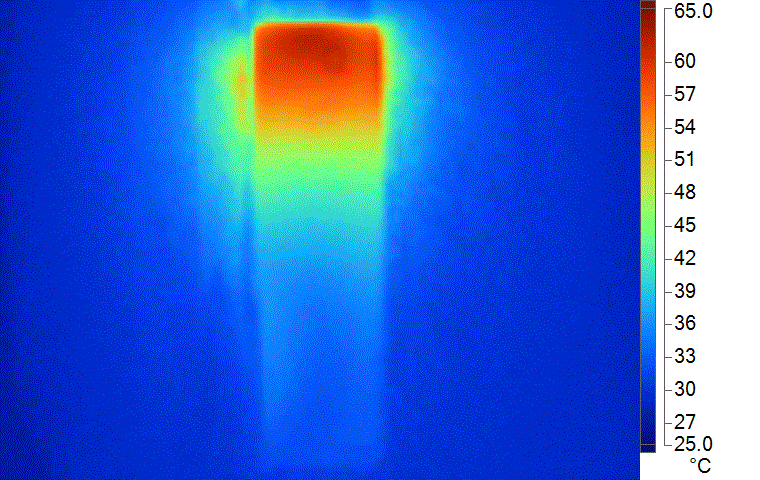

Supplement: Supplementary file 6 — Source Data [file 41467_2023_39190_MOESM6_ESM.zip › 1-original data/fig. 4/fig.4b-up/45s.gif]

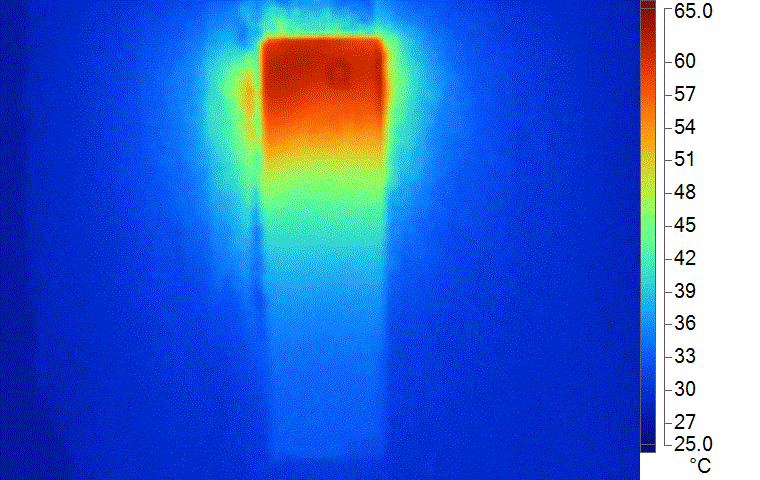

Supplement: Supplementary file 6 — Source Data [file 41467_2023_39190_MOESM6_ESM.zip › 1-original data/fig. 4/fig.4b-up/55s.gif]

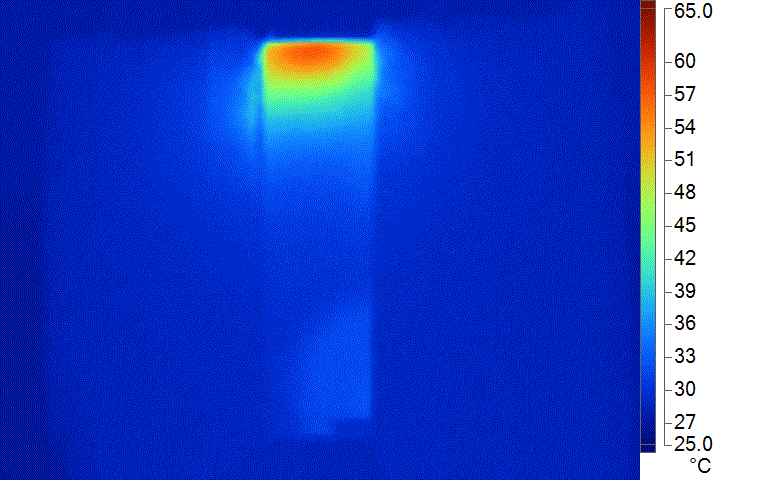

Supplement: Supplementary file 6 — Source Data [file 41467_2023_39190_MOESM6_ESM.zip › 1-original data/fig. 4/fig.4b-up/5s.gif]

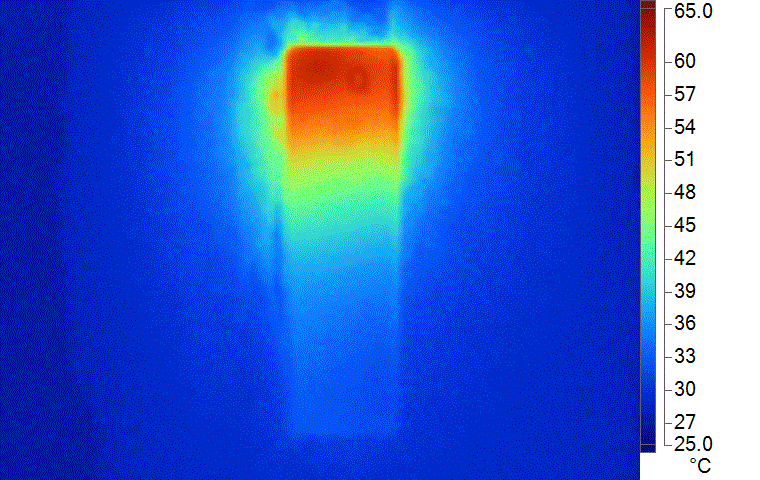

Supplement: Supplementary file 6 — Source Data [file 41467_2023_39190_MOESM6_ESM.zip › 1-original data/fig. 4/fig.4b-up/65s.gif]

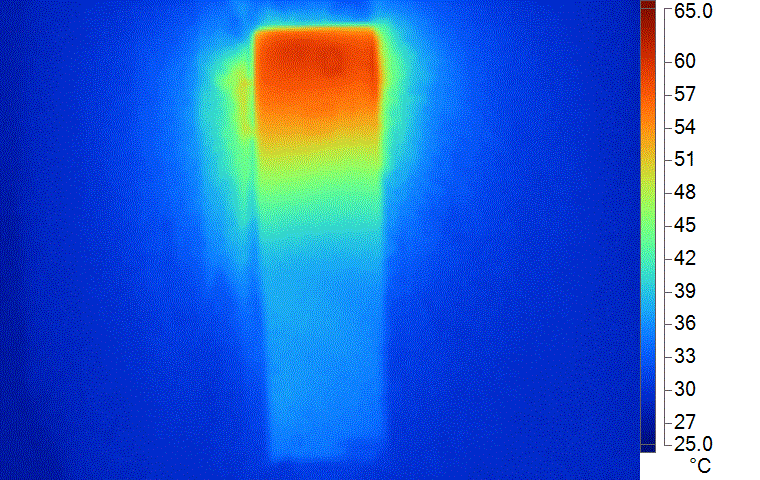

Supplement: Supplementary file 6 — Source Data [file 41467_2023_39190_MOESM6_ESM.zip › 1-original data/fig. 4/fig.4b-up/75s.gif]

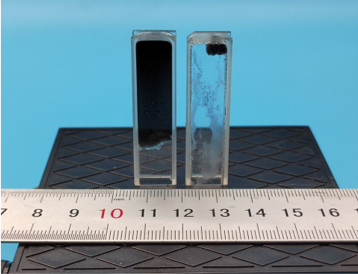

Supplement: Supplementary file 6 — Source Data [file 41467_2023_39190_MOESM6_ESM.zip › 1-original data/fig. 4/Fig.4e.png]

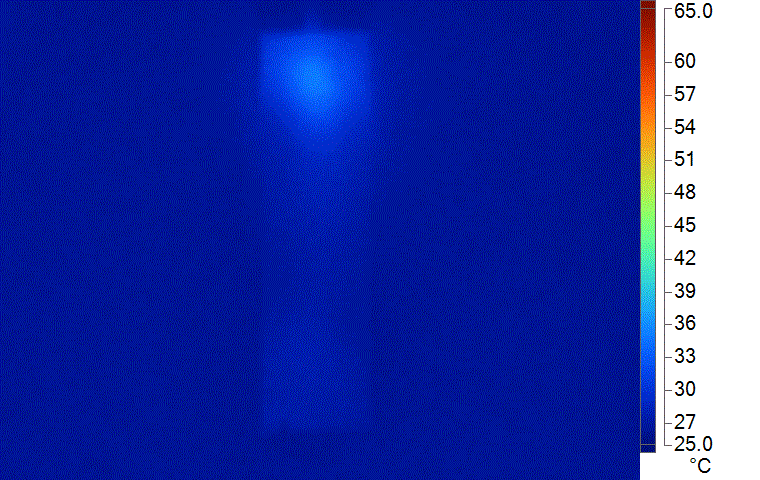

Supplement: Supplementary file 6 — Source Data [file 41467_2023_39190_MOESM6_ESM.zip › 1-original data/fig. 4/fig4b-down/4b-0s.gif]

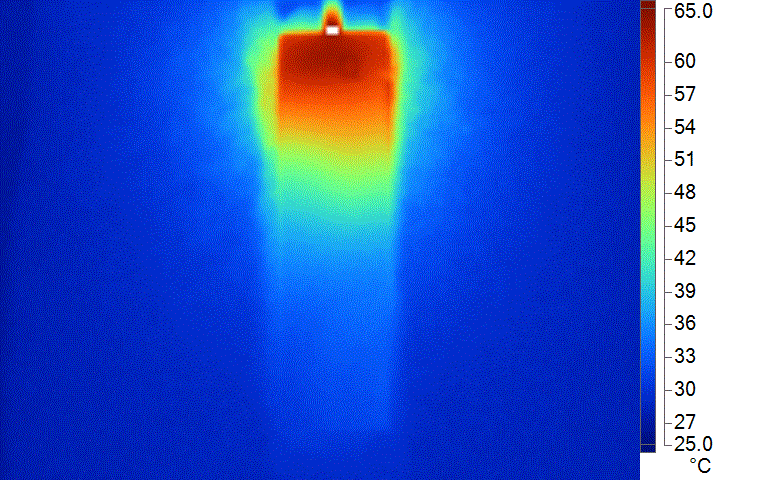

Supplement: Supplementary file 6 — Source Data [file 41467_2023_39190_MOESM6_ESM.zip › 1-original data/fig. 4/fig4b-down/4b-10s.gif]

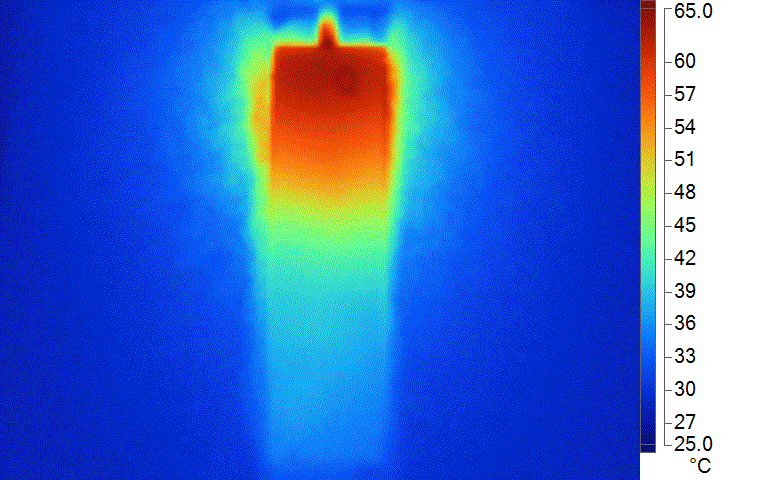

Supplement: Supplementary file 6 — Source Data [file 41467_2023_39190_MOESM6_ESM.zip › 1-original data/fig. 4/fig4b-down/4b-15s.gif]

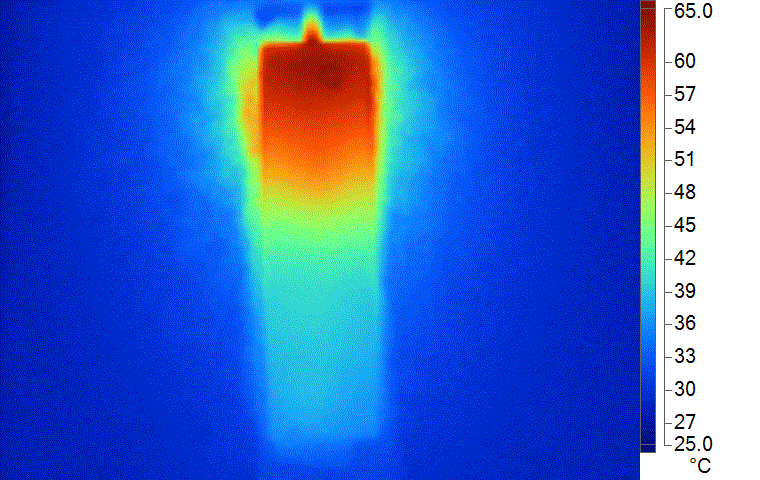

Supplement: Supplementary file 6 — Source Data [file 41467_2023_39190_MOESM6_ESM.zip › 1-original data/fig. 4/fig4b-down/4b-20s.gif]

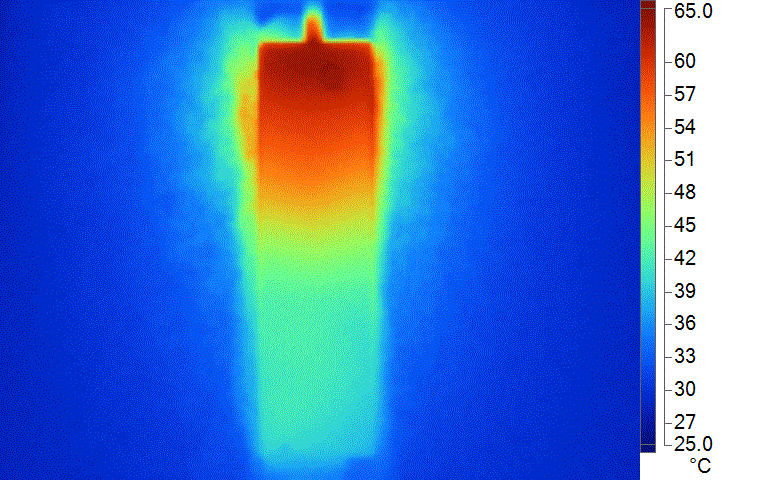

Supplement: Supplementary file 6 — Source Data [file 41467_2023_39190_MOESM6_ESM.zip › 1-original data/fig. 4/fig4b-down/4b-25s.gif]

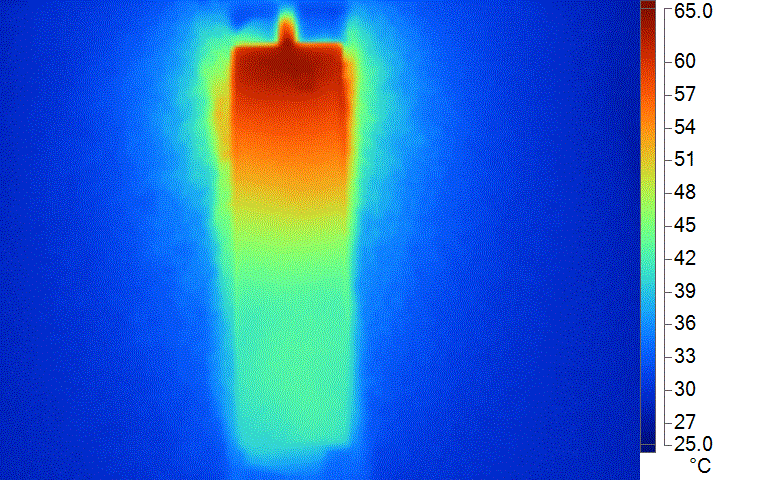

Supplement: Supplementary file 6 — Source Data [file 41467_2023_39190_MOESM6_ESM.zip › 1-original data/fig. 4/fig4b-down/4b-30s.gif]

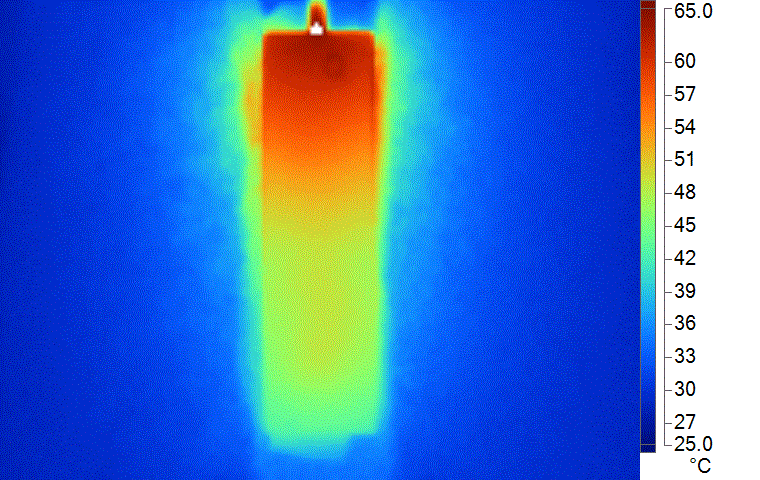

Supplement: Supplementary file 6 — Source Data [file 41467_2023_39190_MOESM6_ESM.zip › 1-original data/fig. 4/fig4b-down/4b-45s.gif]

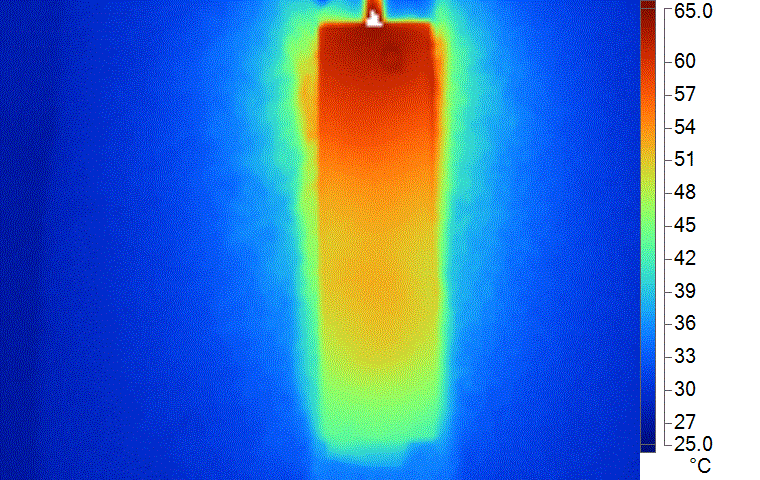

Supplement: Supplementary file 6 — Source Data [file 41467_2023_39190_MOESM6_ESM.zip › 1-original data/fig. 4/fig4b-down/4b-55s.gif]

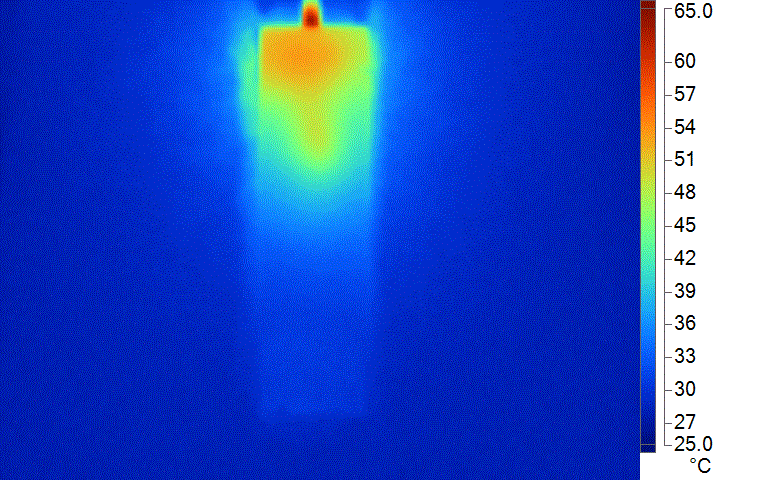

Supplement: Supplementary file 6 — Source Data [file 41467_2023_39190_MOESM6_ESM.zip › 1-original data/fig. 4/fig4b-down/4b-5s.gif]

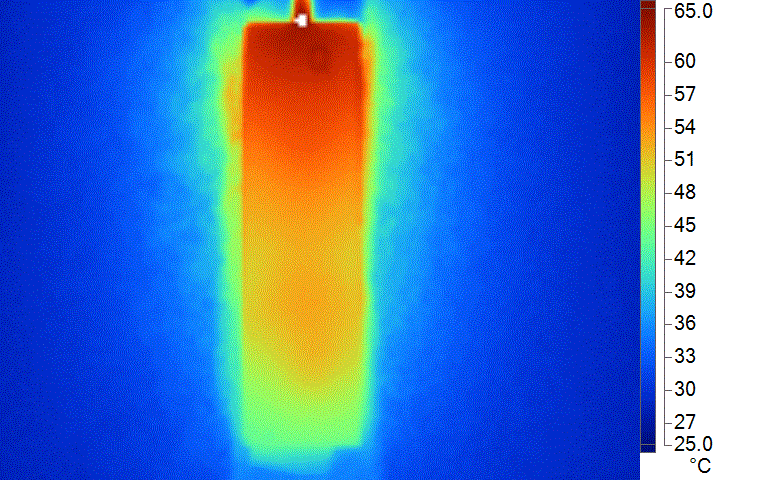

Supplement: Supplementary file 6 — Source Data [file 41467_2023_39190_MOESM6_ESM.zip › 1-original data/fig. 4/fig4b-down/4b-65png.png]

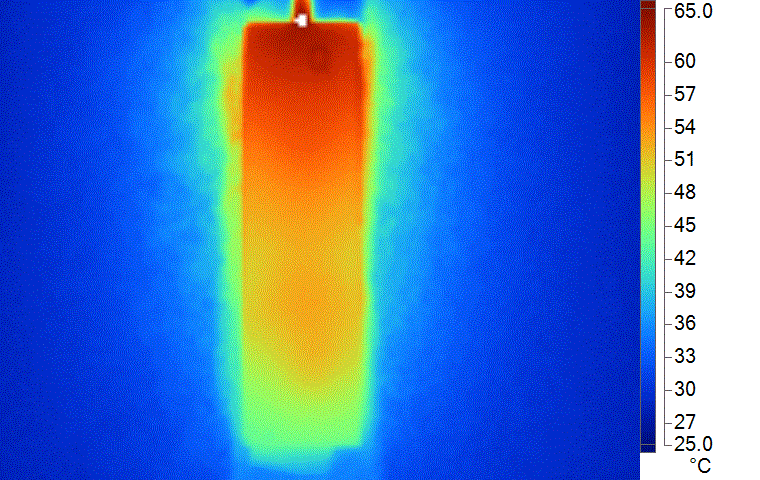

Supplement: Supplementary file 6 — Source Data [file 41467_2023_39190_MOESM6_ESM.zip › 1-original data/fig. 4/fig4b-down/4b-65s.gif]

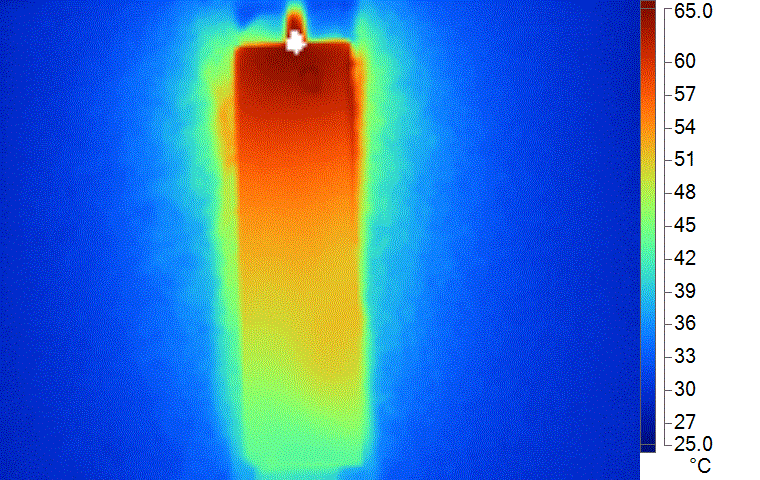

Supplement: Supplementary file 6 — Source Data [file 41467_2023_39190_MOESM6_ESM.zip › 1-original data/fig. 4/fig4b-down/4b-75s.gif]

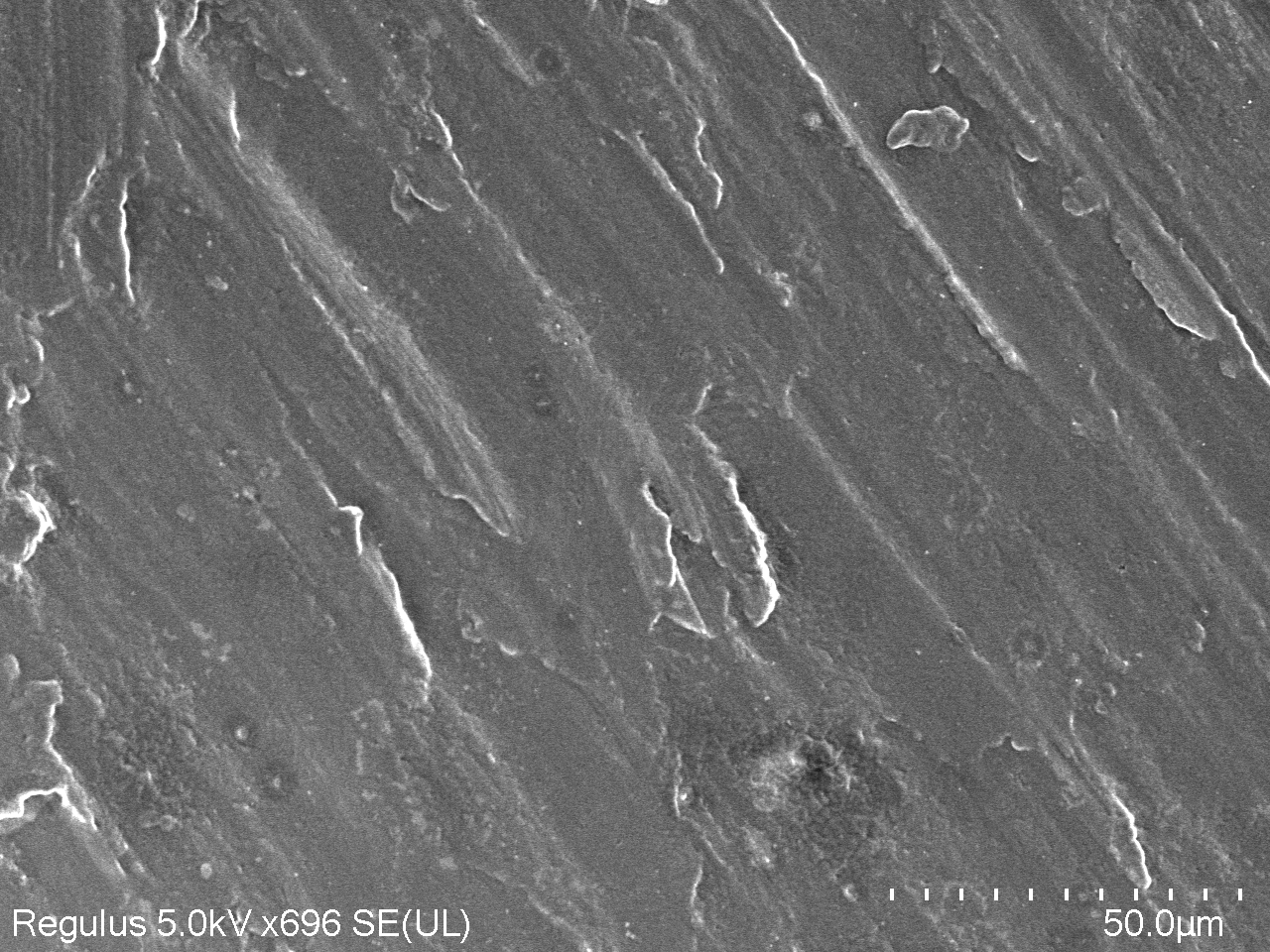

Supplement: Supplementary file 6 — Source Data [file 41467_2023_39190_MOESM6_ESM.zip › 1-original data/Fig.2/Fig.2a.tif]

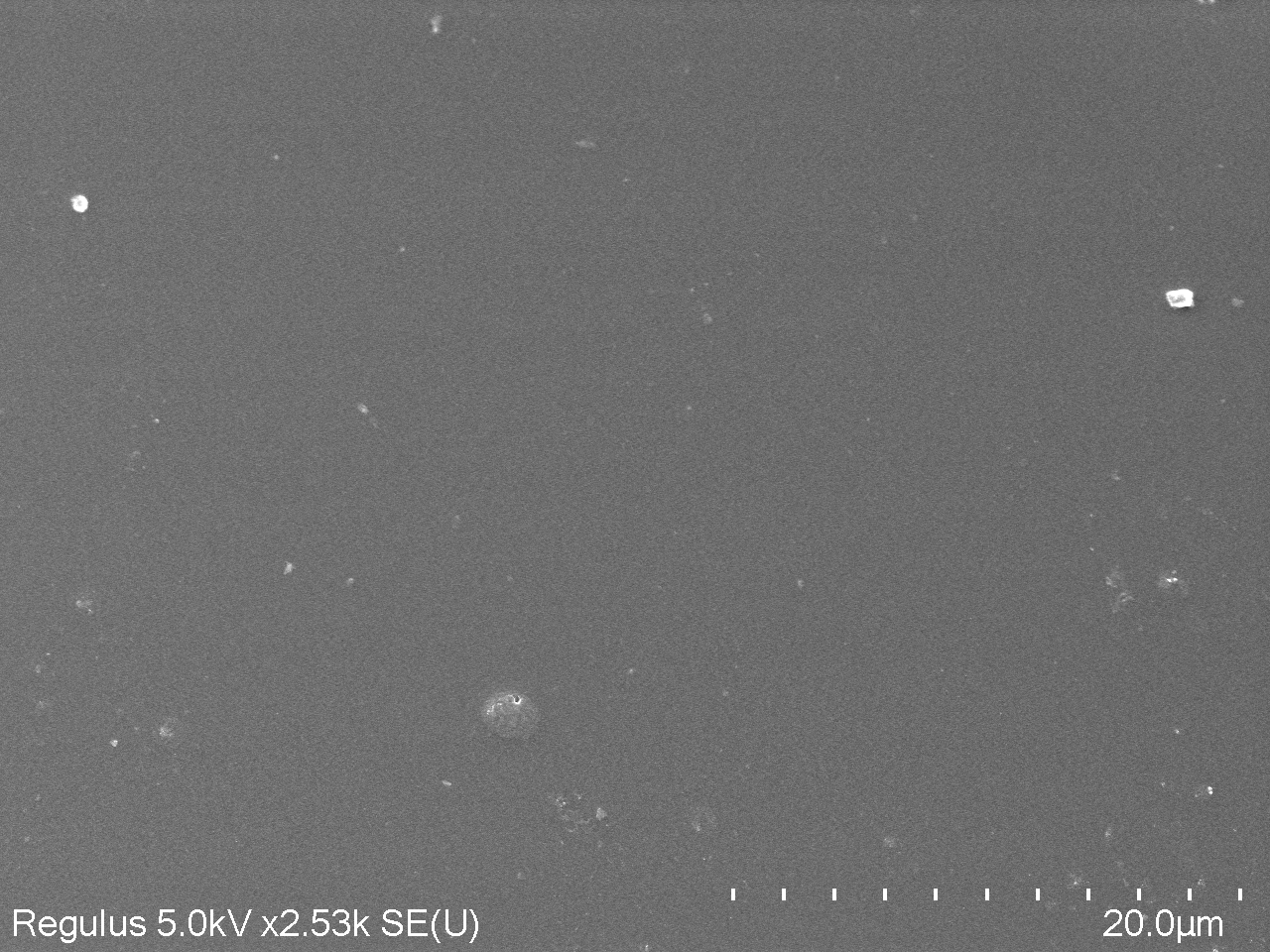

Supplement: Supplementary file 6 — Source Data [file 41467_2023_39190_MOESM6_ESM.zip › 1-original data/Fig.3/Fig. 3c.tif]

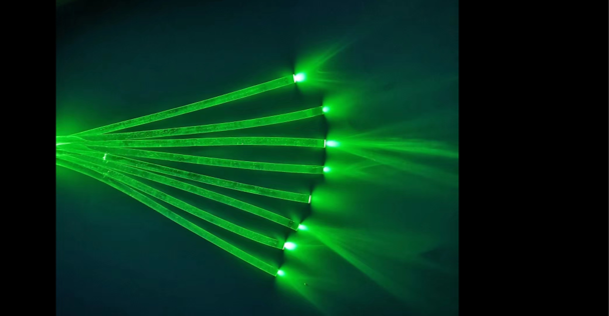

Supplement: Supplementary file 6 — Source Data [file 41467_2023_39190_MOESM6_ESM.zip › 1-original data/Fig.3/Fig.3a.png]

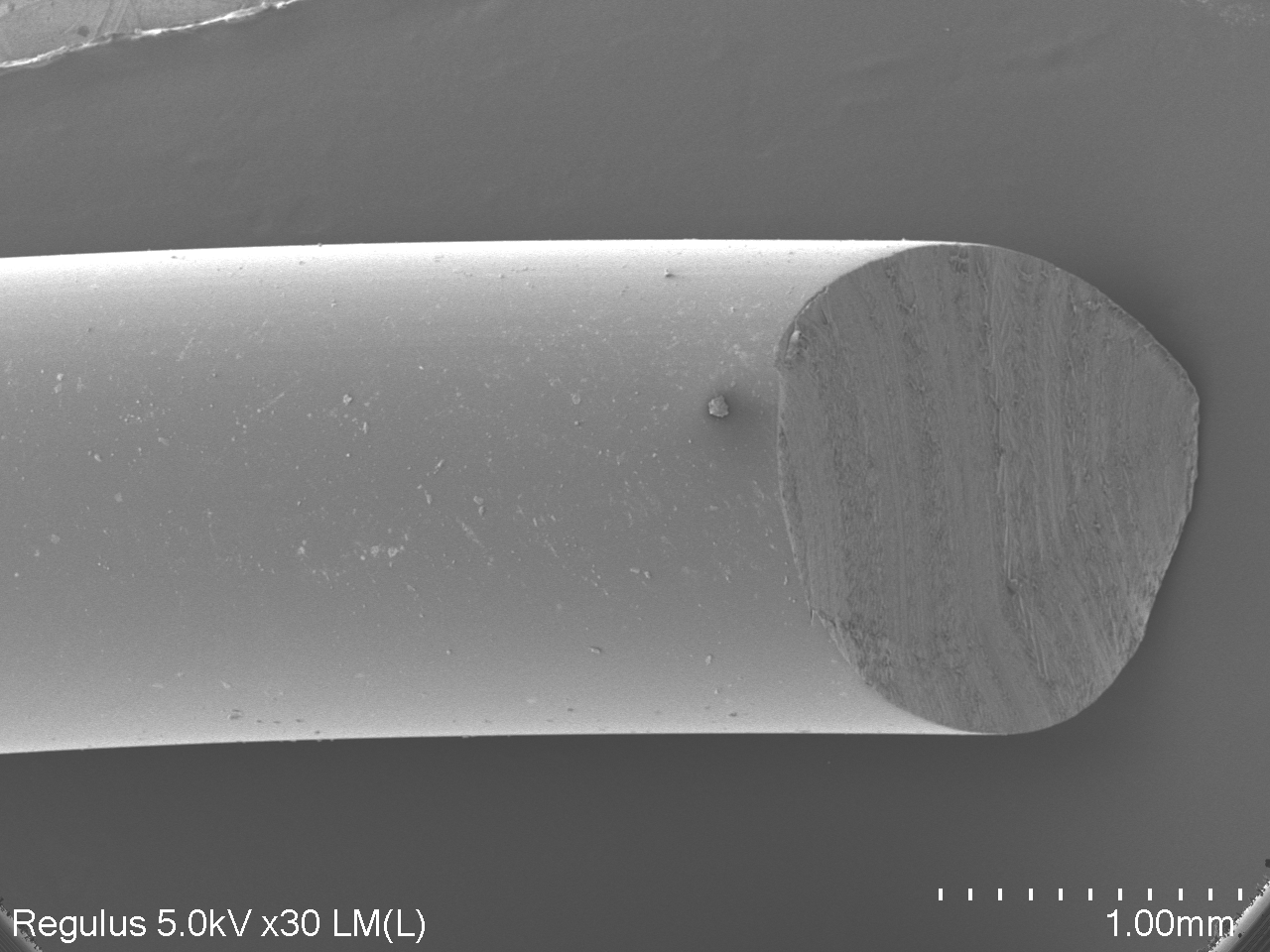

Supplement: Supplementary file 6 — Source Data [file 41467_2023_39190_MOESM6_ESM.zip › 1-original data/Fig.3/Fig.3b.tif]

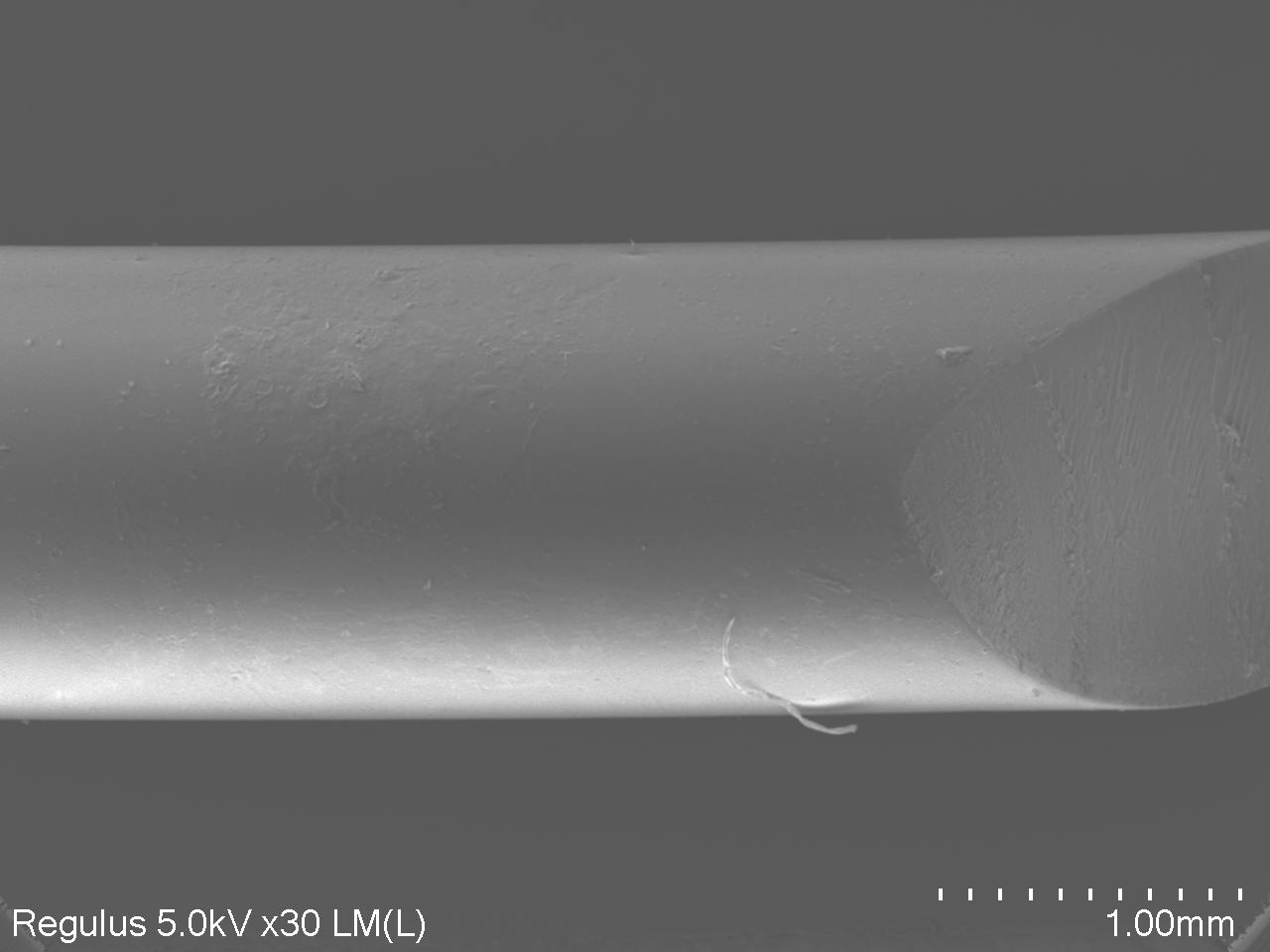

Supplement: Supplementary file 6 — Source Data [file 41467_2023_39190_MOESM6_ESM.zip › 1-original data/Fig.3/Fig.3d.tif]

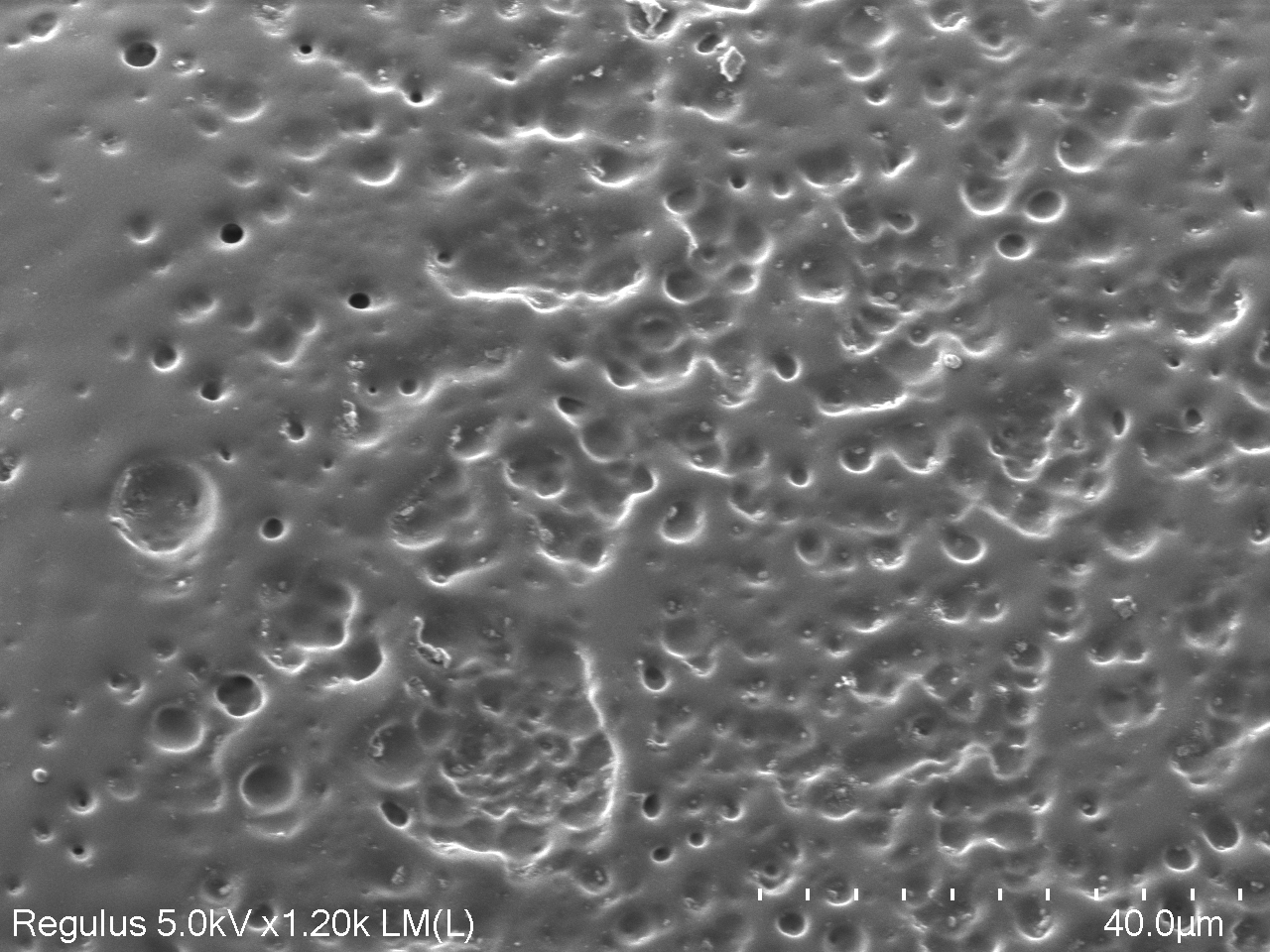

Supplement: Supplementary file 6 — Source Data [file 41467_2023_39190_MOESM6_ESM.zip › 1-original data/Fig.3/Fig.3e.tif]

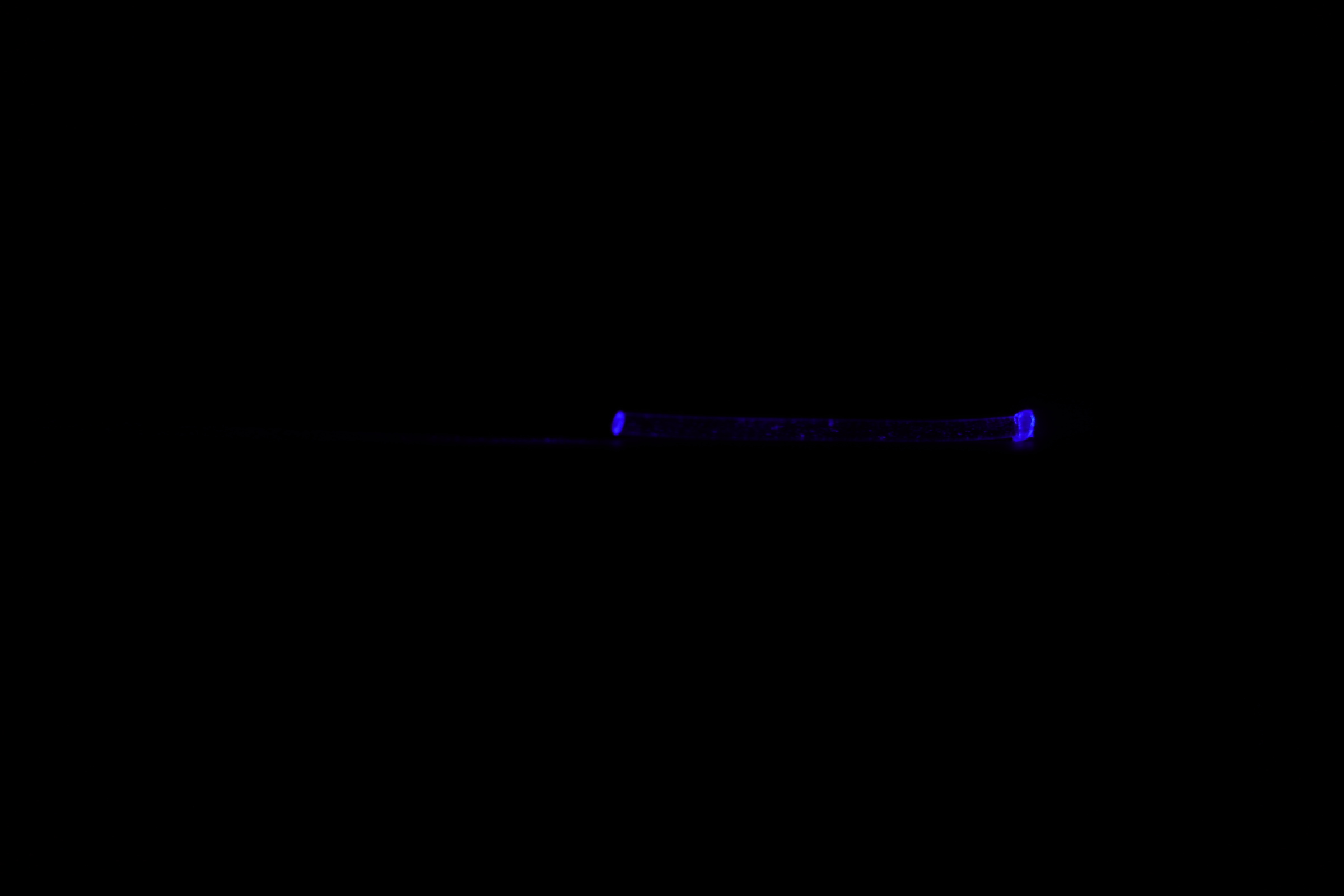

Supplement: Supplementary file 6 — Source Data [file 41467_2023_39190_MOESM6_ESM.zip › 1-original data/Fig.3/Fig.3f.JPG]

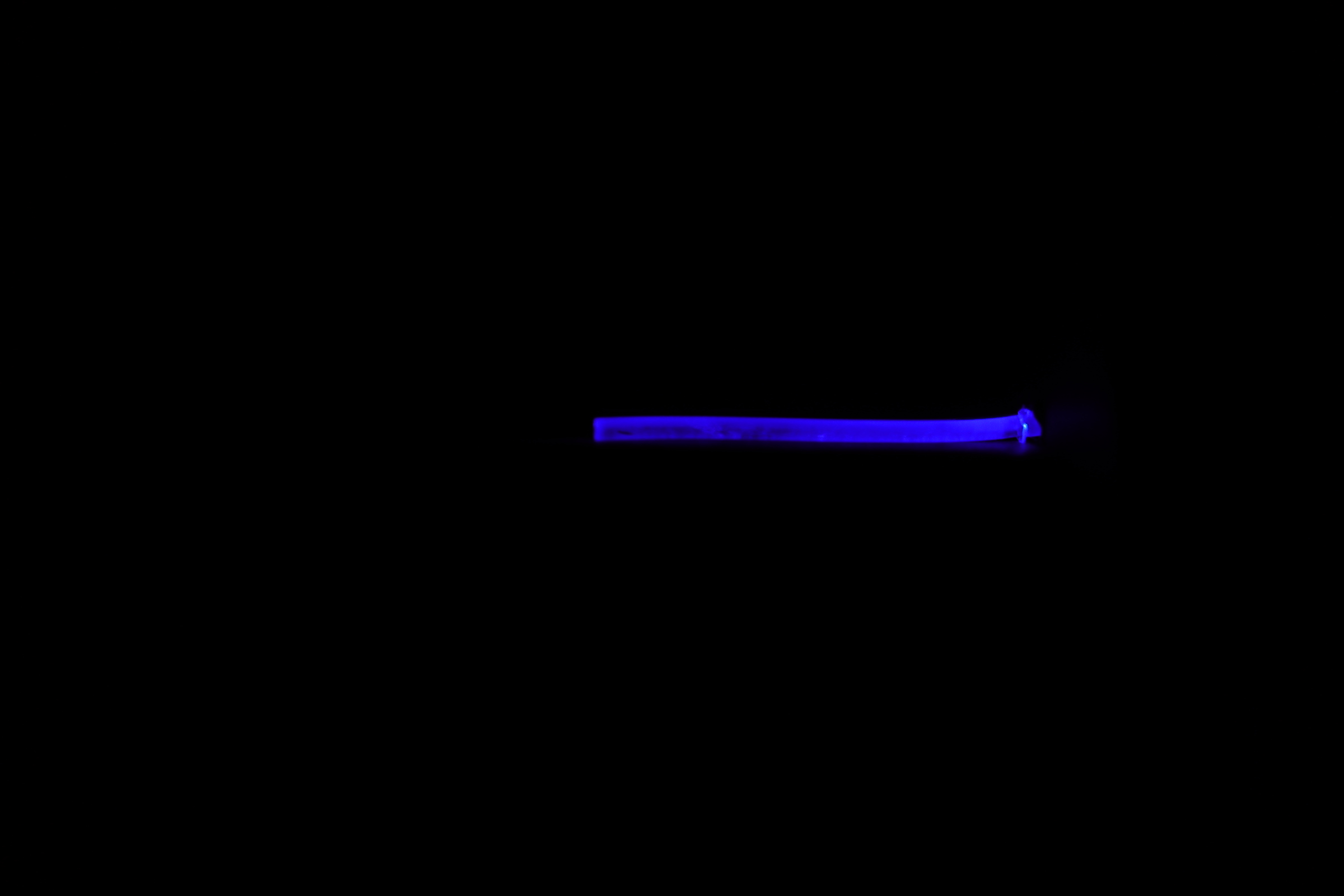

Supplement: Supplementary file 6 — Source Data [file 41467_2023_39190_MOESM6_ESM.zip › 1-original data/Fig.3/Fig.3g.JPG]

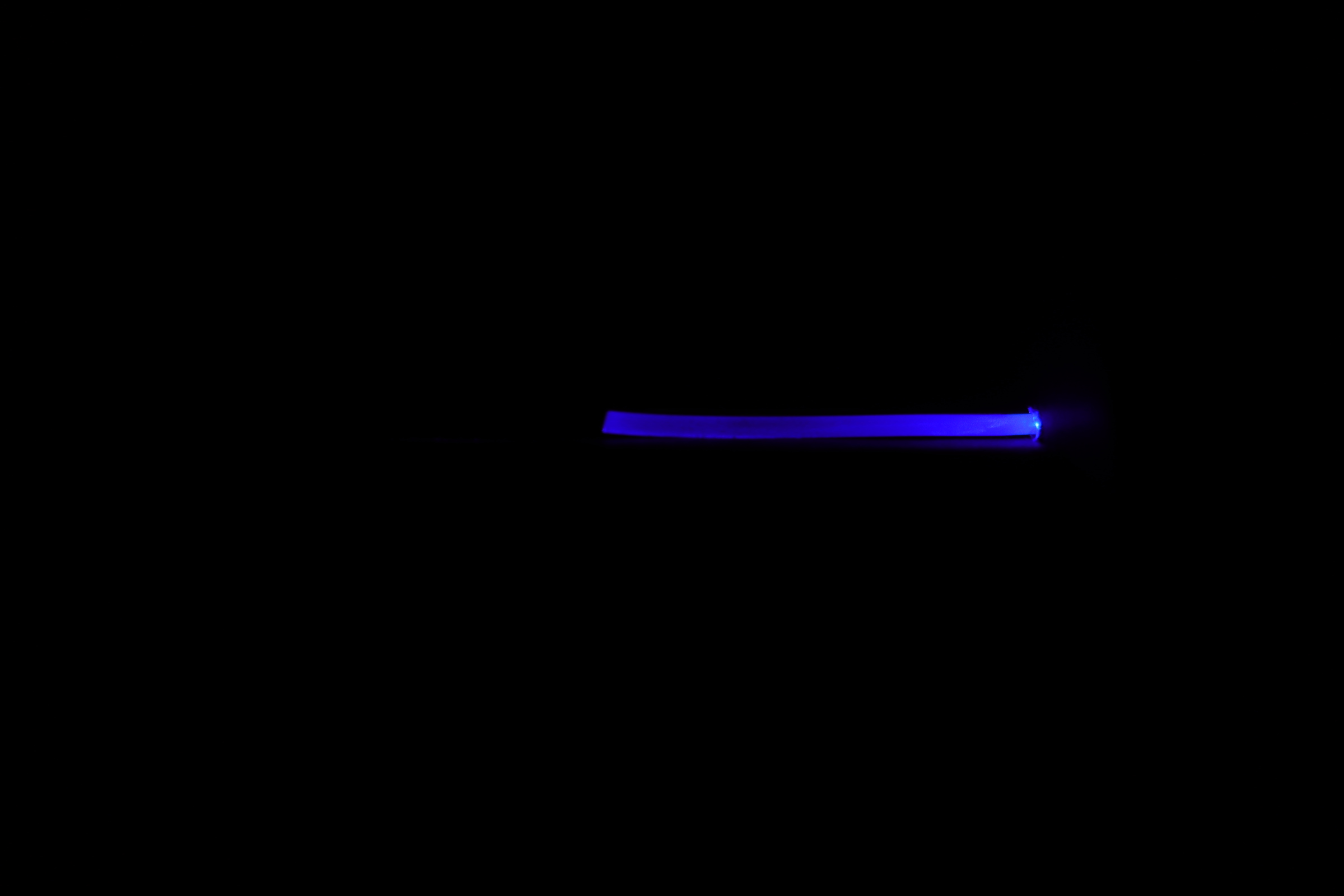

Supplement: Supplementary file 6 — Source Data [file 41467_2023_39190_MOESM6_ESM.zip › 1-original data/Fig.3/Fig.3h/15s.JPG]

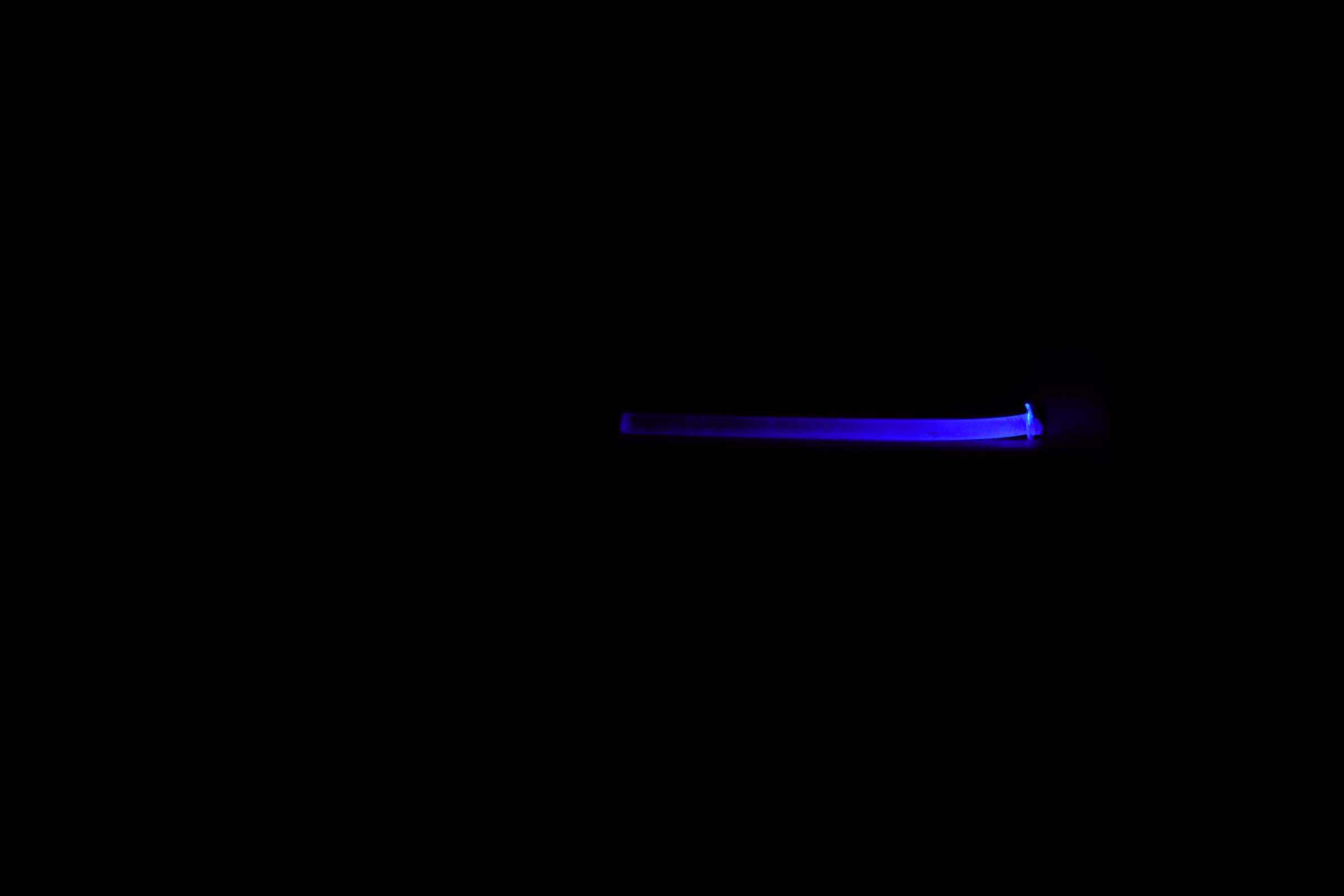

Supplement: Supplementary file 6 — Source Data [file 41467_2023_39190_MOESM6_ESM.zip › 1-original data/Fig.3/Fig.3h/20s.JPG]

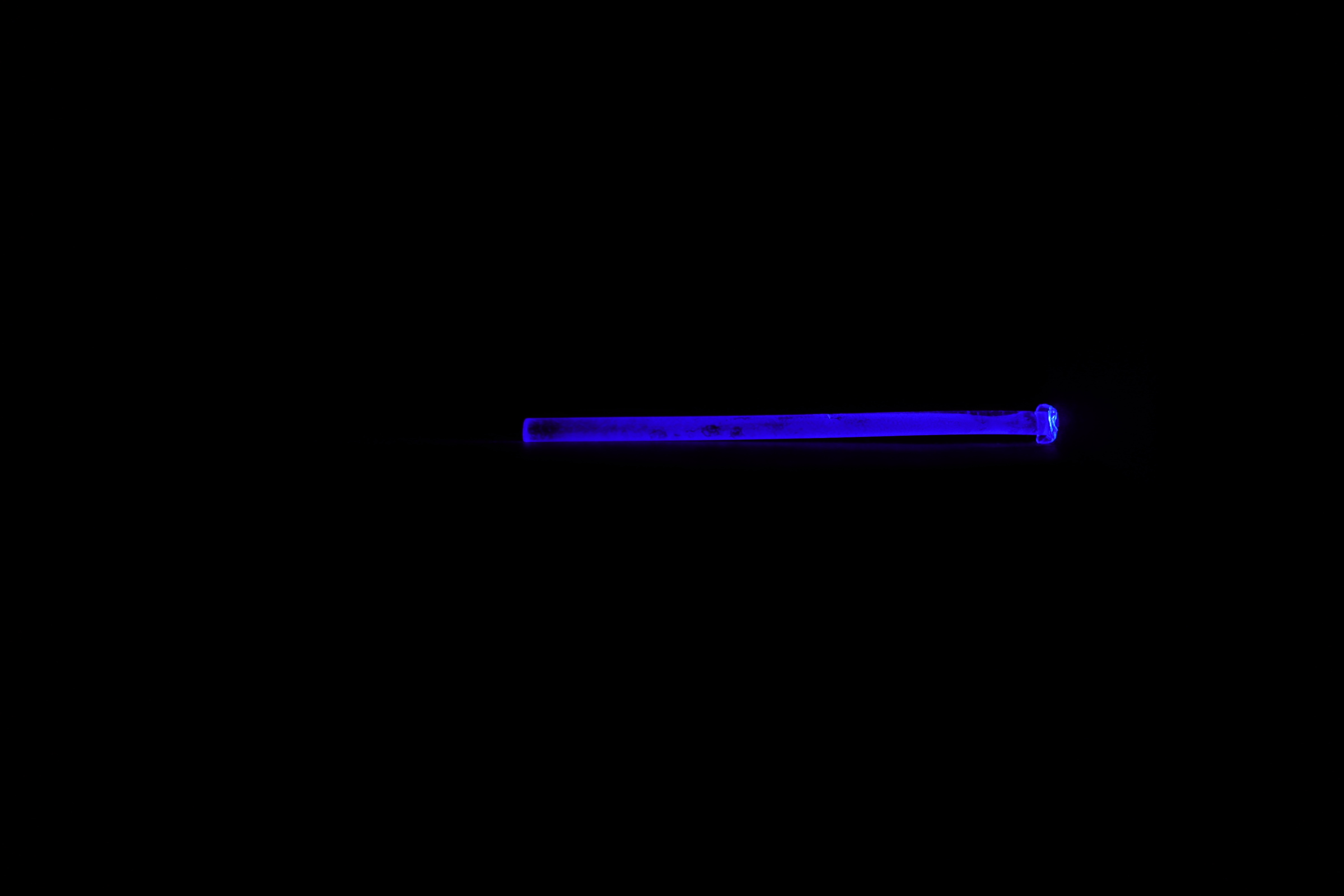

Supplement: Supplementary file 6 — Source Data [file 41467_2023_39190_MOESM6_ESM.zip › 1-original data/Fig.3/Fig.3h/3s.JPG]

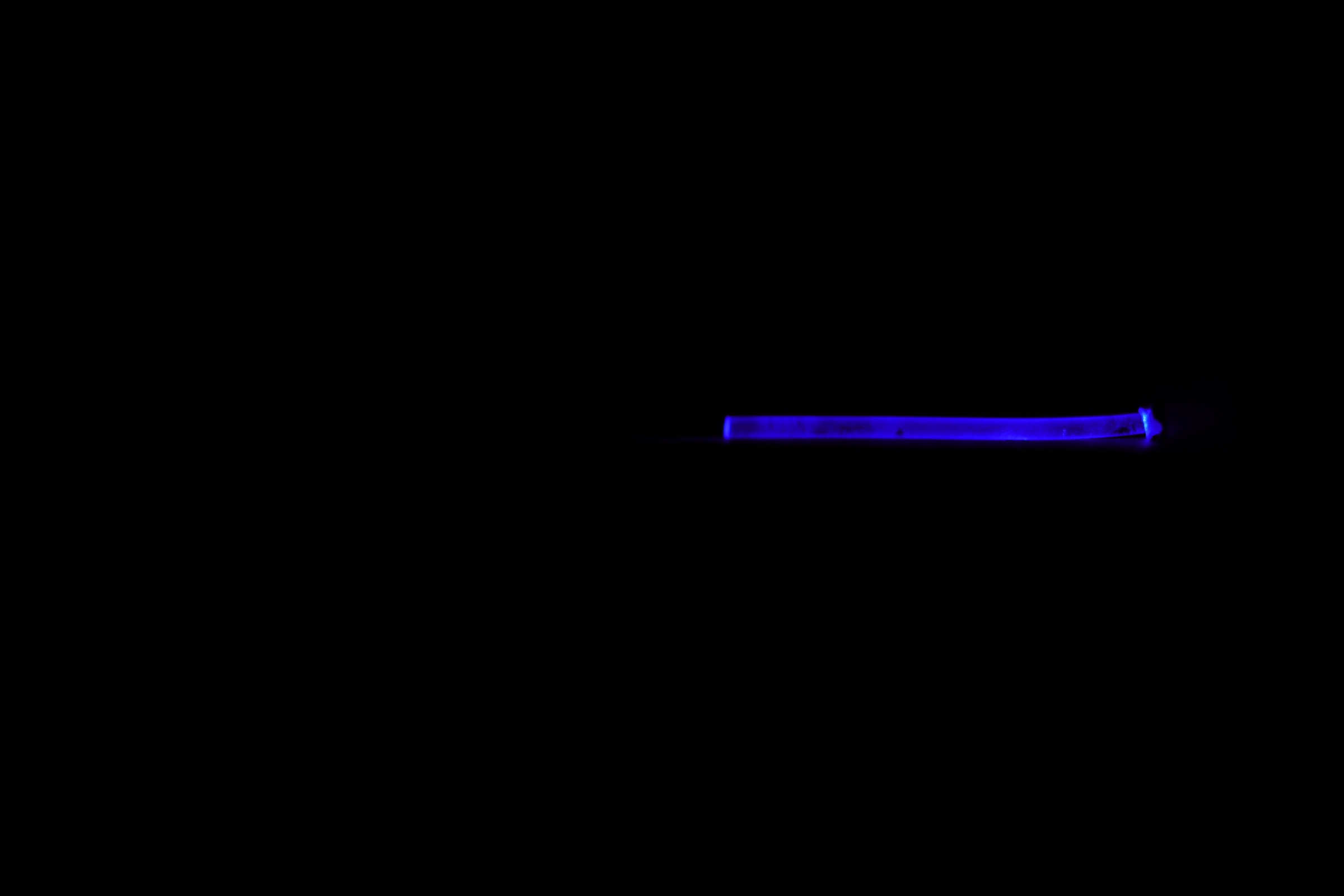

Supplement: Supplementary file 6 — Source Data [file 41467_2023_39190_MOESM6_ESM.zip › 1-original data/Fig.3/Fig.3h/5s.JPG]

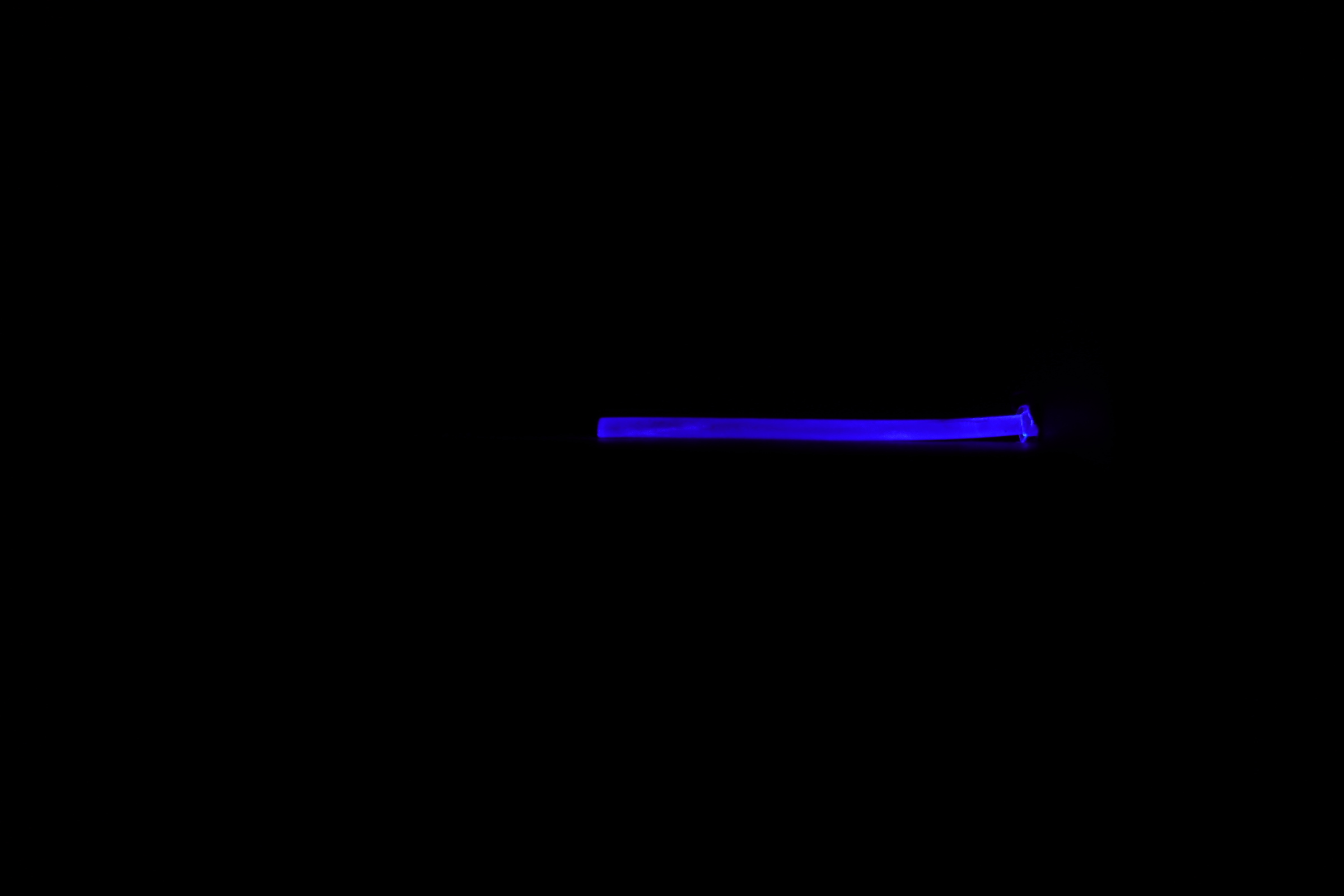

Supplement: Supplementary file 6 — Source Data [file 41467_2023_39190_MOESM6_ESM.zip › 1-original data/Fig.3/Fig.3h/7s.JPG]

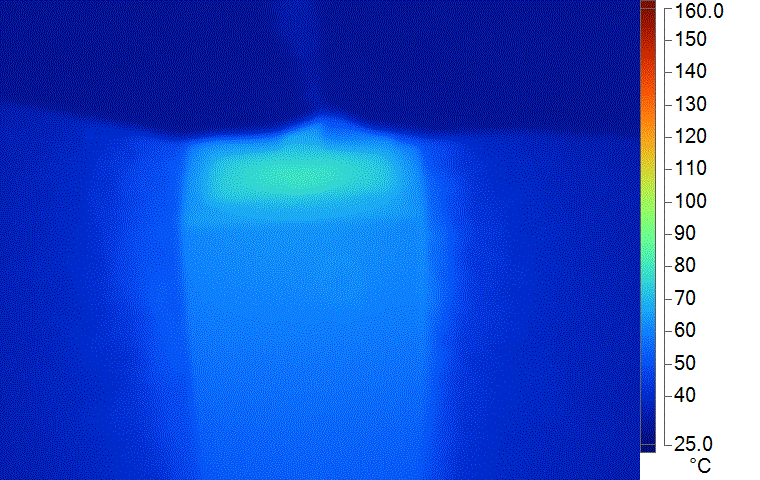

Supplement: Supplementary file 6 — Source Data [file 41467_2023_39190_MOESM6_ESM.zip › 1-original data/Fig.5/Fig, 5b.gif]

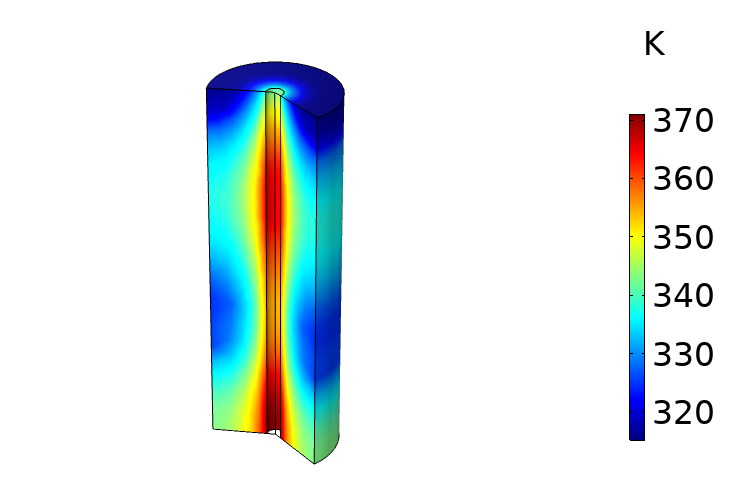

Supplement: Supplementary file 6 — Source Data [file 41467_2023_39190_MOESM6_ESM.zip › 1-original data/Fig.5/Fig, 5f.png]

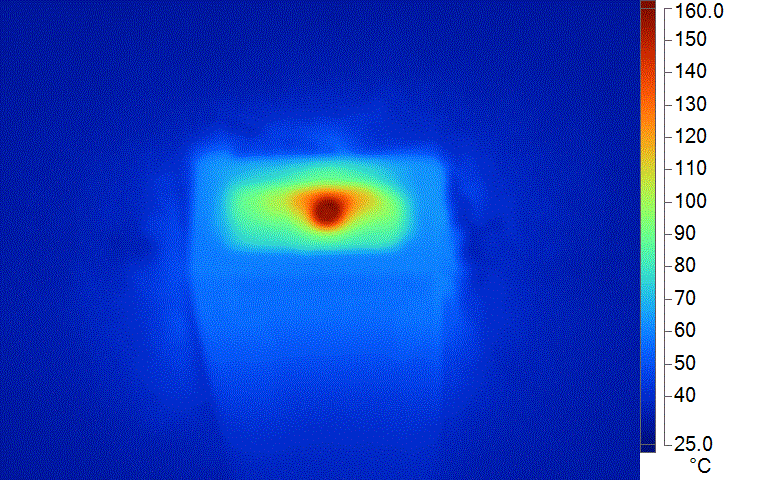

Supplement: Supplementary file 6 — Source Data [file 41467_2023_39190_MOESM6_ESM.zip › 1-original data/Fig.5/Fig. 5a.gif]

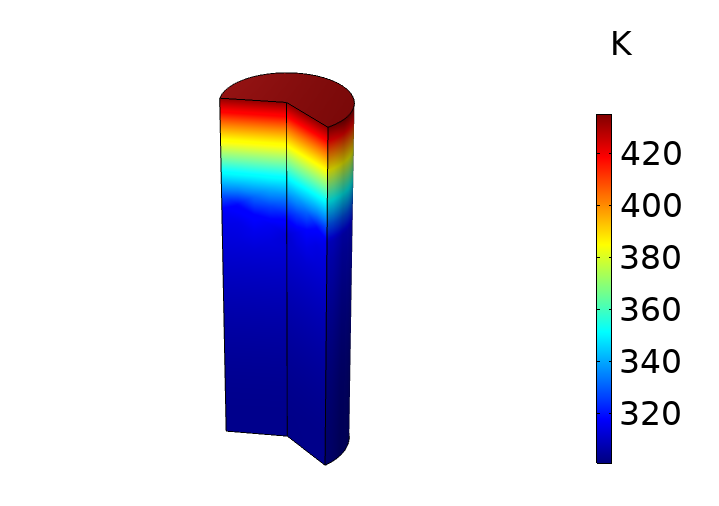

Supplement: Supplementary file 6 — Source Data [file 41467_2023_39190_MOESM6_ESM.zip › 1-original data/Fig.5/Fig.5e.png]

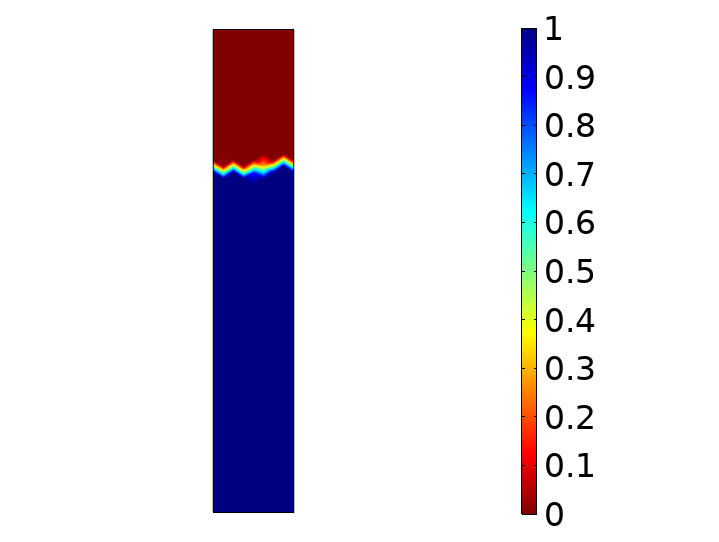

Supplement: Supplementary file 6 — Source Data [file 41467_2023_39190_MOESM6_ESM.zip › 1-original data/Fig.5/Fig.5g/1500s 相指示.png]

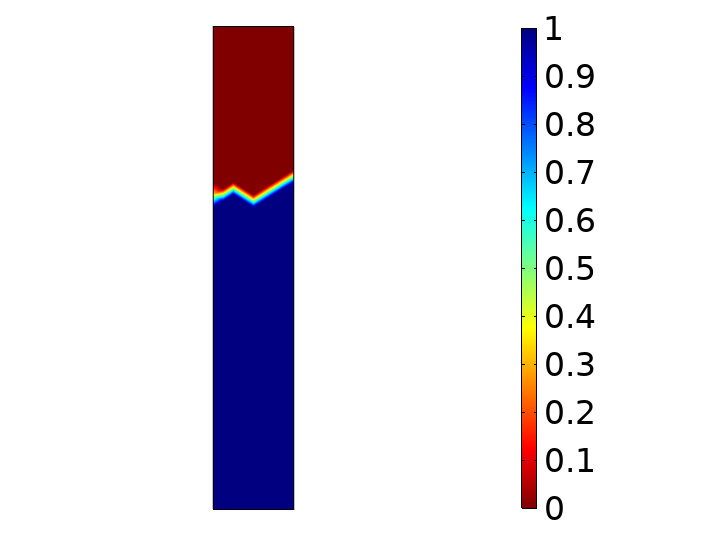

Supplement: Supplementary file 6 — Source Data [file 41467_2023_39190_MOESM6_ESM.zip › 1-original data/Fig.5/Fig.5g/2190 相指示.png]

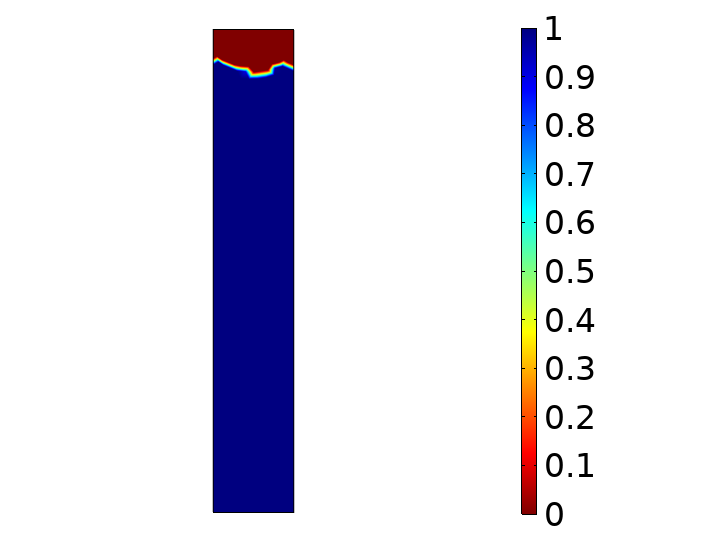

Supplement: Supplementary file 6 — Source Data [file 41467_2023_39190_MOESM6_ESM.zip › 1-original data/Fig.5/Fig.5g/300s 相指示.png]

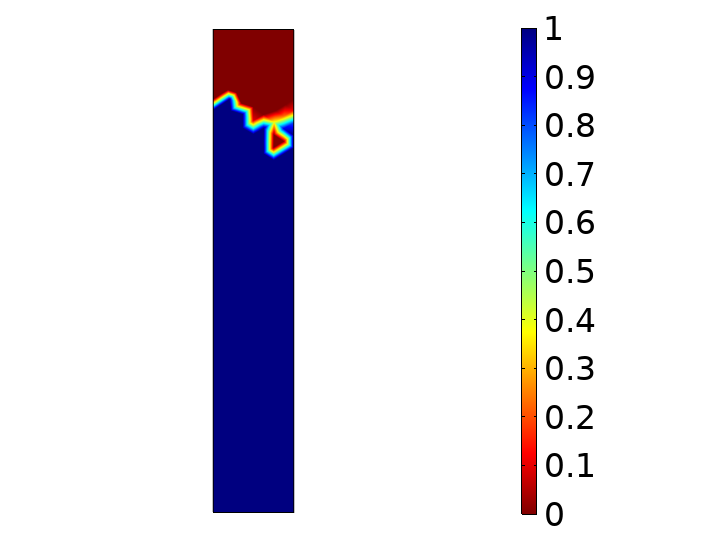

Supplement: Supplementary file 6 — Source Data [file 41467_2023_39190_MOESM6_ESM.zip › 1-original data/Fig.5/Fig.5g/900s 相指示.png]

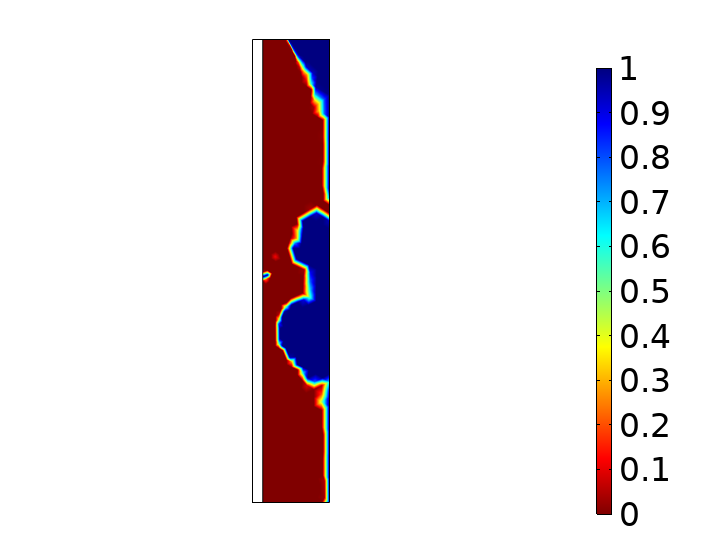

Supplement: Supplementary file 6 — Source Data [file 41467_2023_39190_MOESM6_ESM.zip › 1-original data/Fig.5/Fig.5h/光波导-1500s相指示器.png]

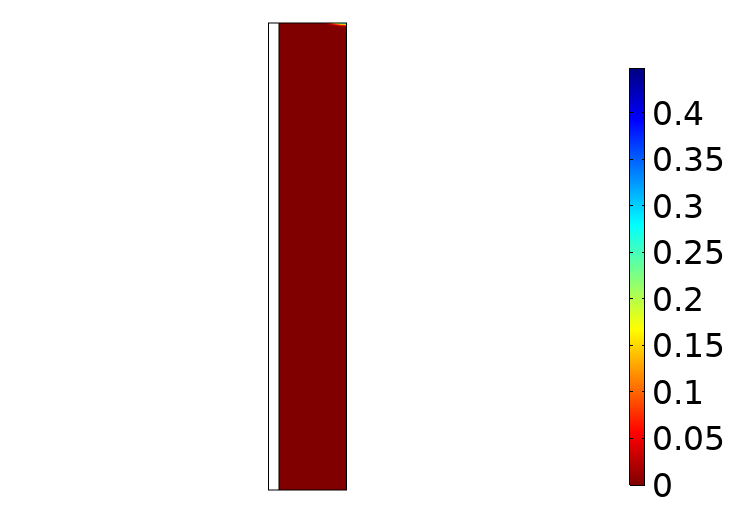

Supplement: Supplementary file 6 — Source Data [file 41467_2023_39190_MOESM6_ESM.zip › 1-original data/Fig.5/Fig.5h/光波导-2190s等相指示器.png]

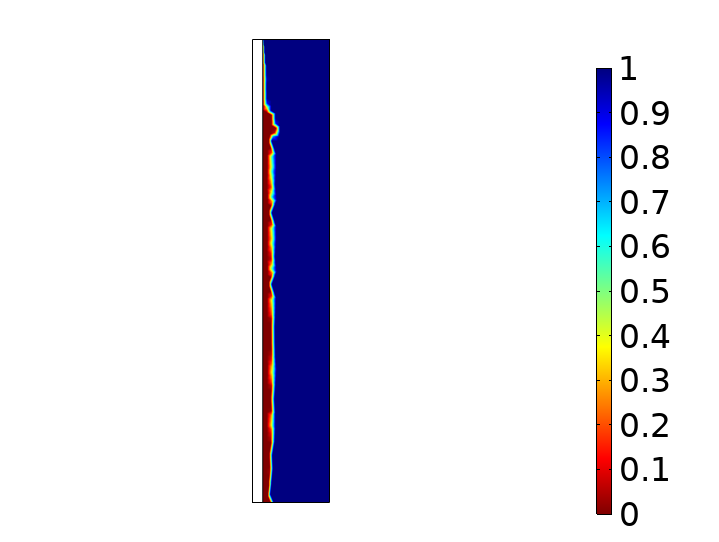

Supplement: Supplementary file 6 — Source Data [file 41467_2023_39190_MOESM6_ESM.zip › 1-original data/Fig.5/Fig.5h/光波导-300s相指示器.png]

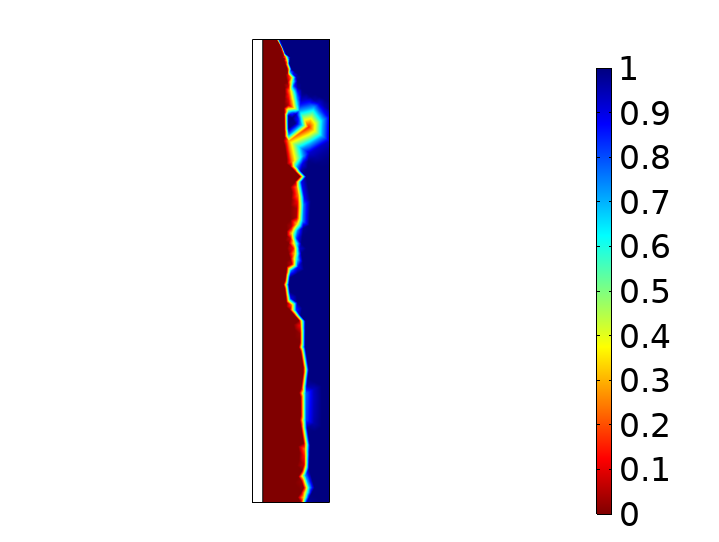

Supplement: Supplementary file 6 — Source Data [file 41467_2023_39190_MOESM6_ESM.zip › 1-original data/Fig.5/Fig.5h/光波导-900s相指示器.png]

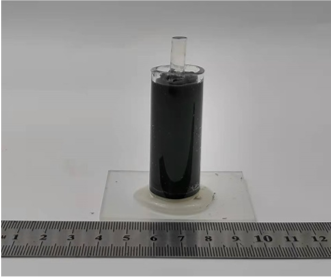

Supplement: Supplementary file 6 — Source Data [file 41467_2023_39190_MOESM6_ESM.zip › 1-original data/fig.6/Fig, 6b.png]

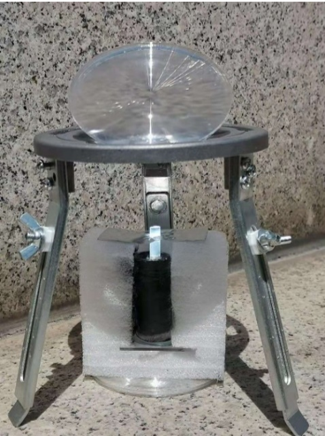

Supplement: Supplementary file 6 — Source Data [file 41467_2023_39190_MOESM6_ESM.zip › 1-original data/fig.6/Fig. 6a.png]

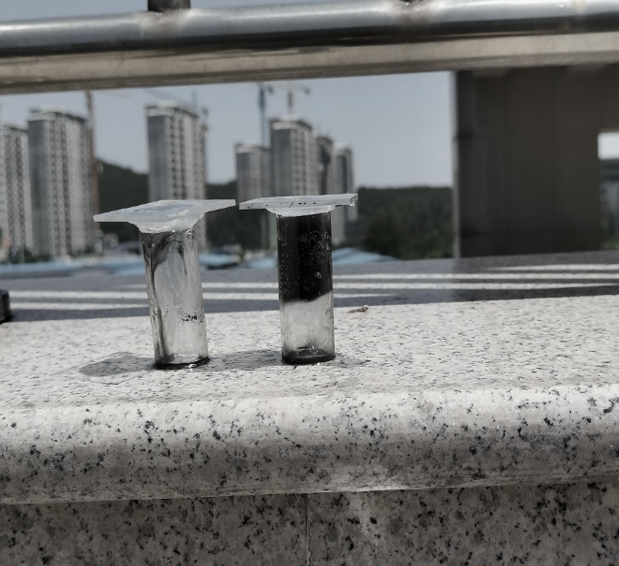

Supplement: Supplementary file 6 — Source Data [file 41467_2023_39190_MOESM6_ESM.zip › 1-original data/fig.6/Fig. 6f&g.png]

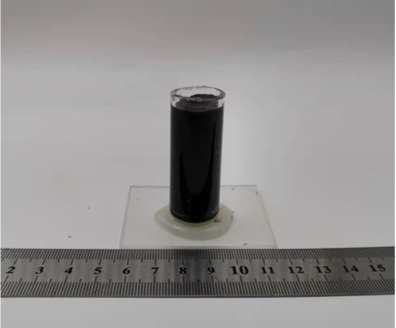

Supplement: Supplementary file 6 — Source Data [file 41467_2023_39190_MOESM6_ESM.zip › 1-original data/fig.6/Fig.6 c.png]

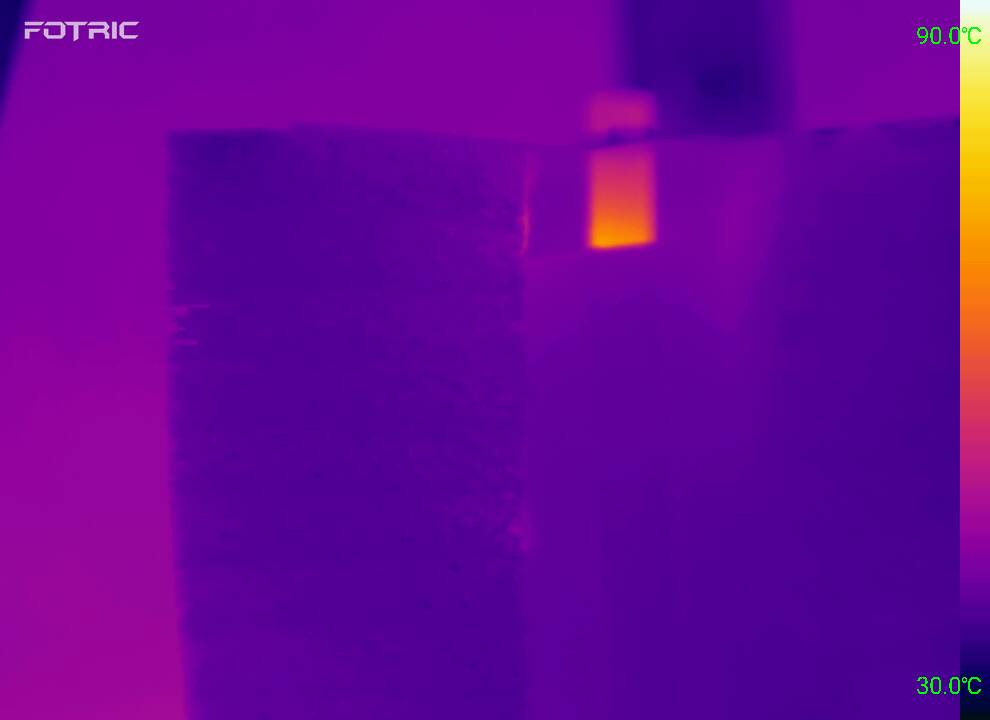

Supplement: Supplementary file 6 — Source Data [file 41467_2023_39190_MOESM6_ESM.zip › 1-original data/fig.6/fig.6e-down/1000.jpg]

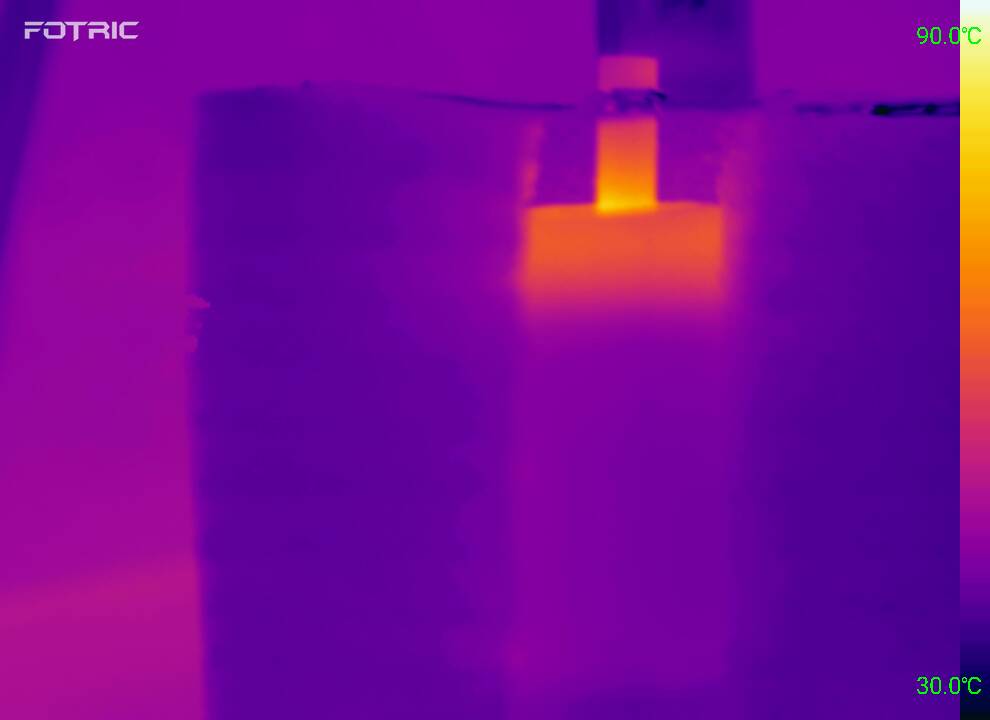

Supplement: Supplementary file 6 — Source Data [file 41467_2023_39190_MOESM6_ESM.zip › 1-original data/fig.6/fig.6e-down/1010.jpg]

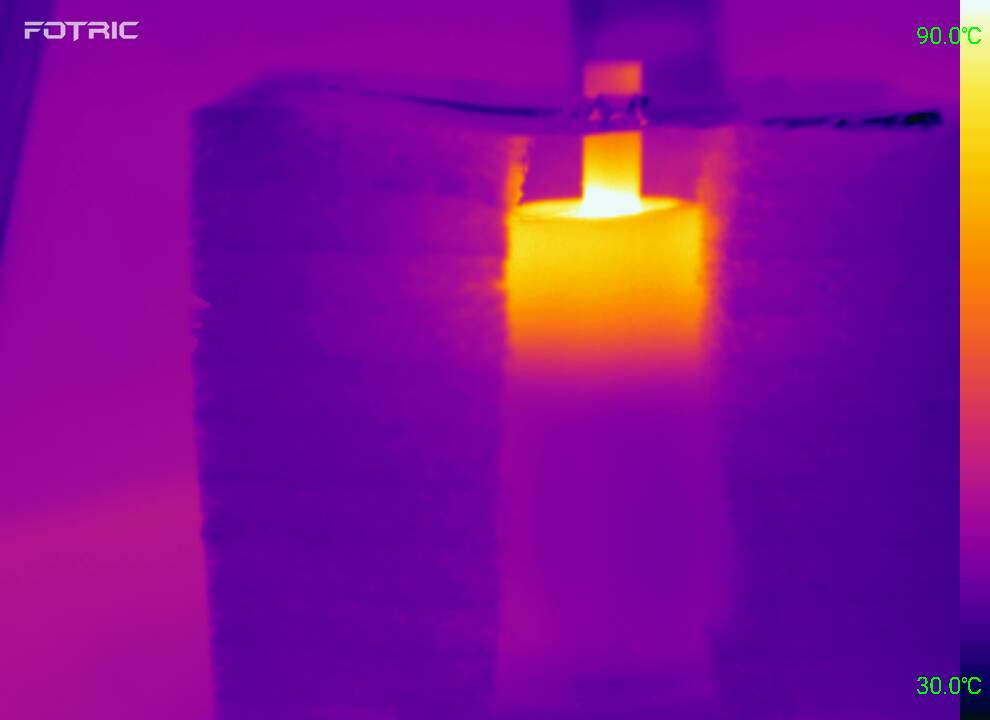

Supplement: Supplementary file 6 — Source Data [file 41467_2023_39190_MOESM6_ESM.zip › 1-original data/fig.6/fig.6e-down/1020.jpg]

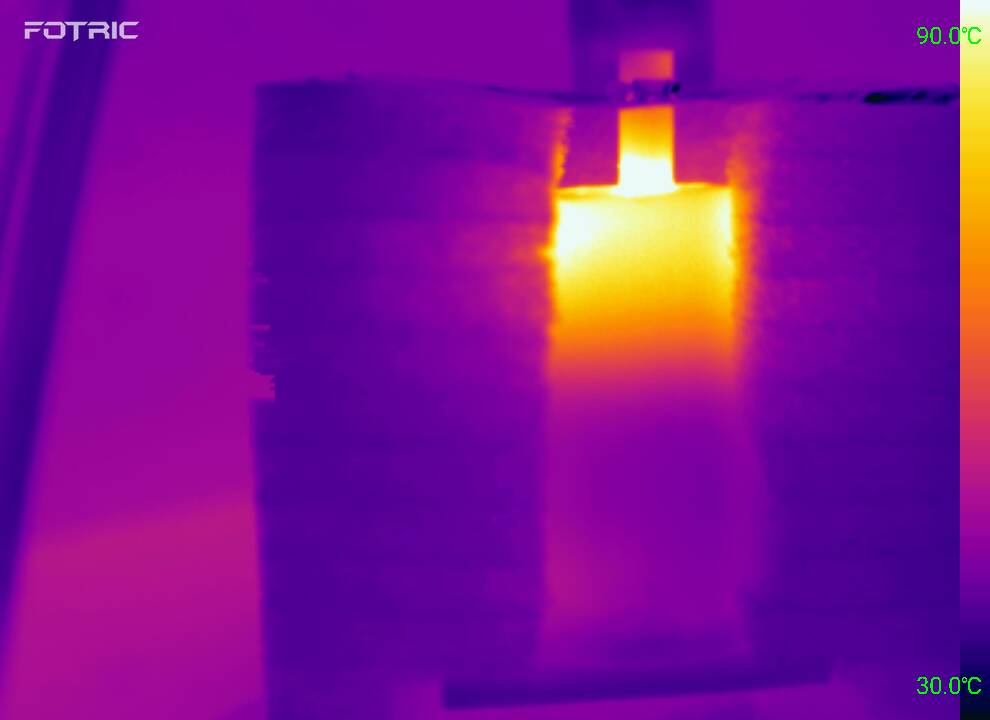

Supplement: Supplementary file 6 — Source Data [file 41467_2023_39190_MOESM6_ESM.zip › 1-original data/fig.6/fig.6e-down/1030.jpg]

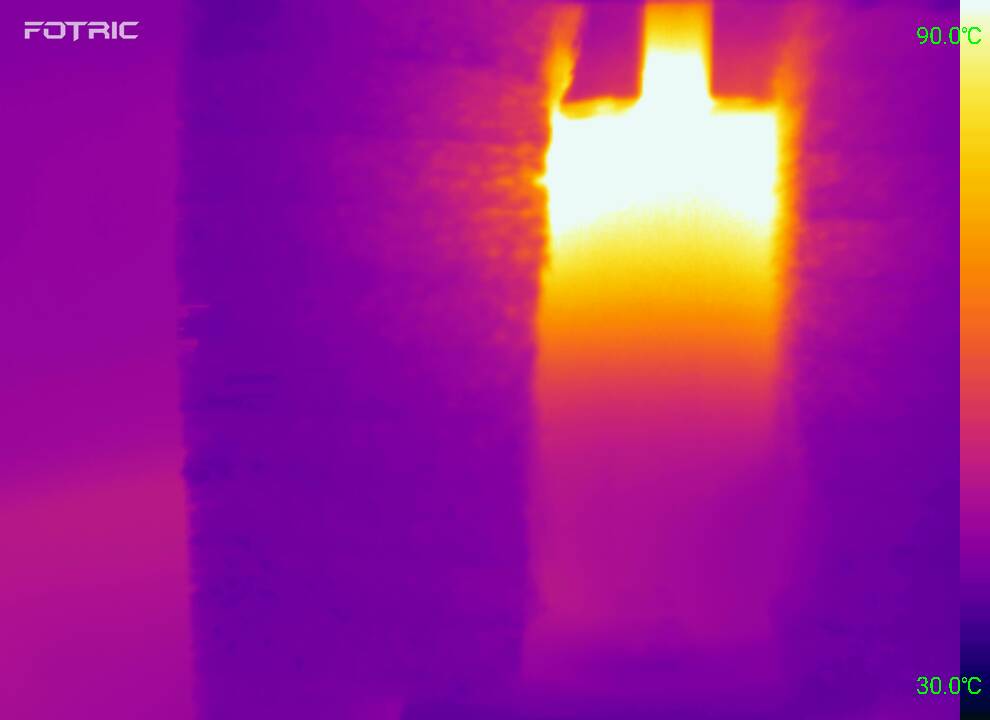

Supplement: Supplementary file 6 — Source Data [file 41467_2023_39190_MOESM6_ESM.zip › 1-original data/fig.6/fig.6e-down/1045.jpg]

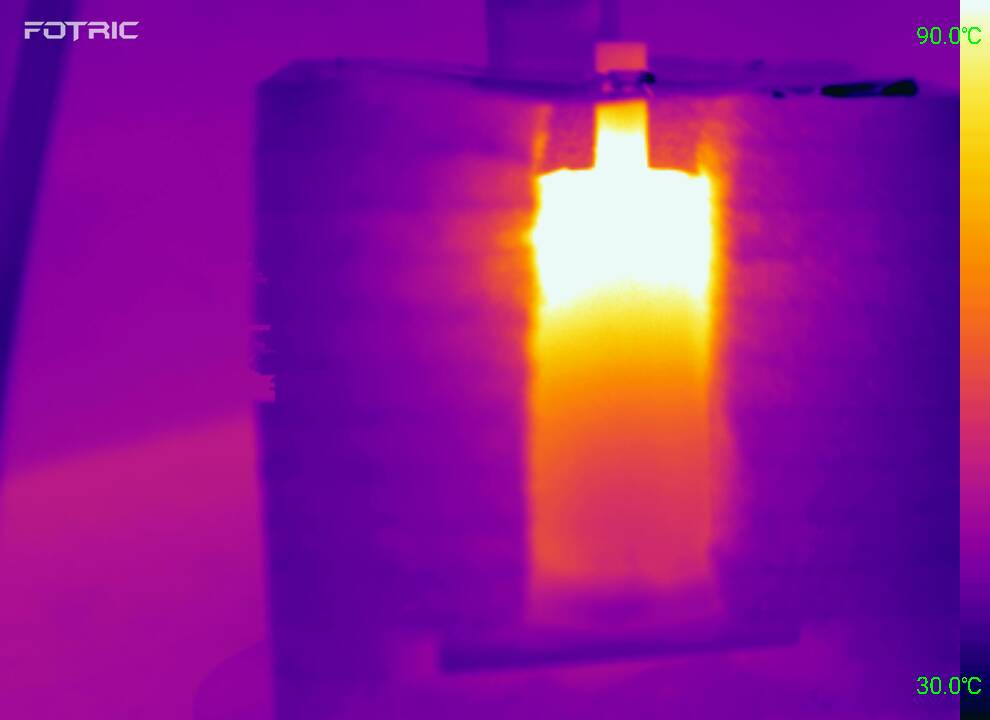

Supplement: Supplementary file 6 — Source Data [file 41467_2023_39190_MOESM6_ESM.zip › 1-original data/fig.6/fig.6e-down/1105.jpg]

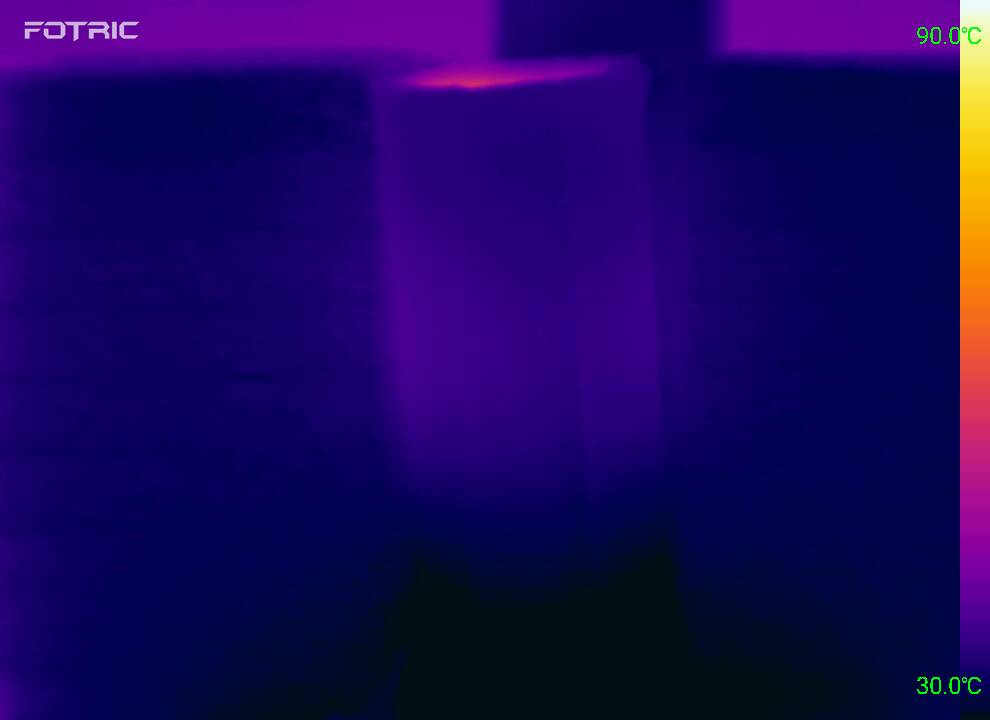

Supplement: Supplementary file 6 — Source Data [file 41467_2023_39190_MOESM6_ESM.zip › 1-original data/fig.6/fig.6e-up/1000.jpg]

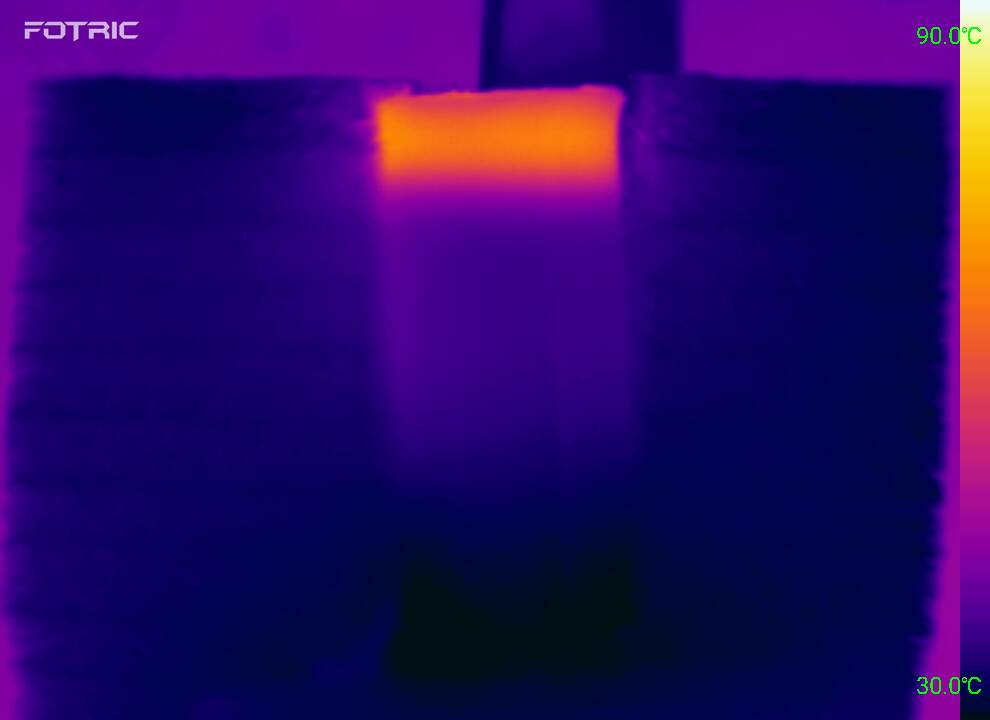

Supplement: Supplementary file 6 — Source Data [file 41467_2023_39190_MOESM6_ESM.zip › 1-original data/fig.6/fig.6e-up/1010.jpg]

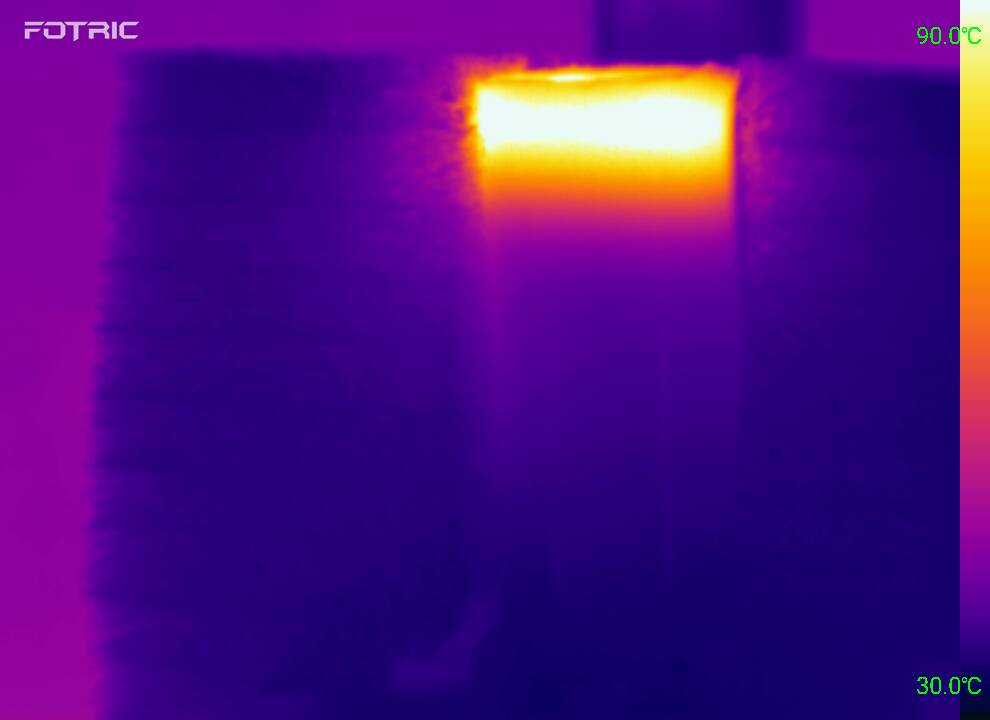

Supplement: Supplementary file 6 — Source Data [file 41467_2023_39190_MOESM6_ESM.zip › 1-original data/fig.6/fig.6e-up/1020.jpg]

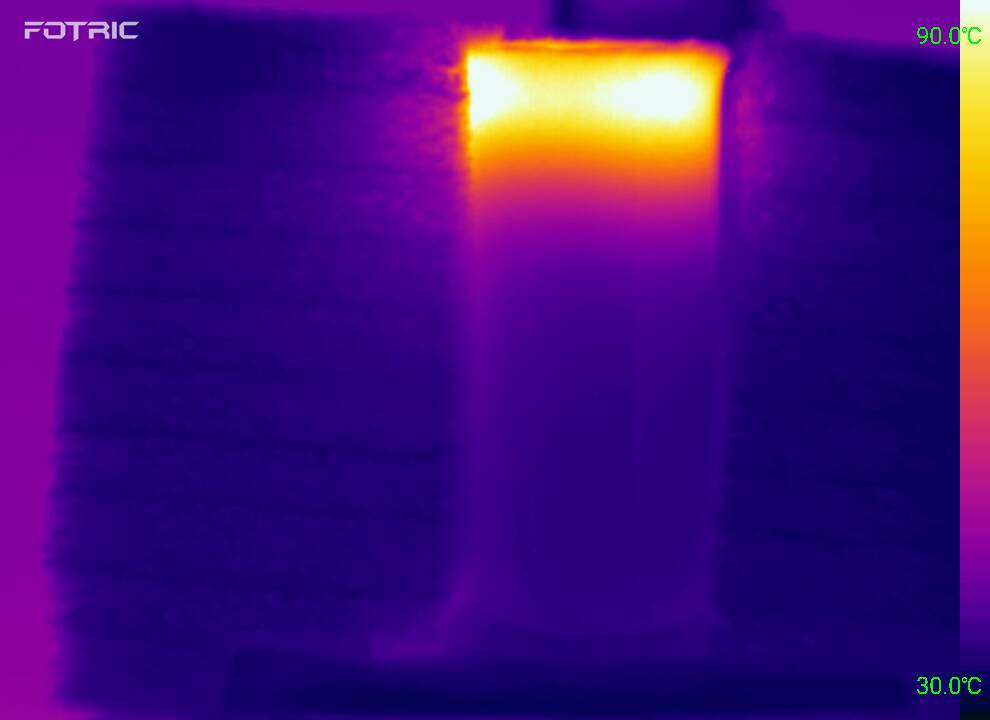

Supplement: Supplementary file 6 — Source Data [file 41467_2023_39190_MOESM6_ESM.zip › 1-original data/fig.6/fig.6e-up/1030.jpg]

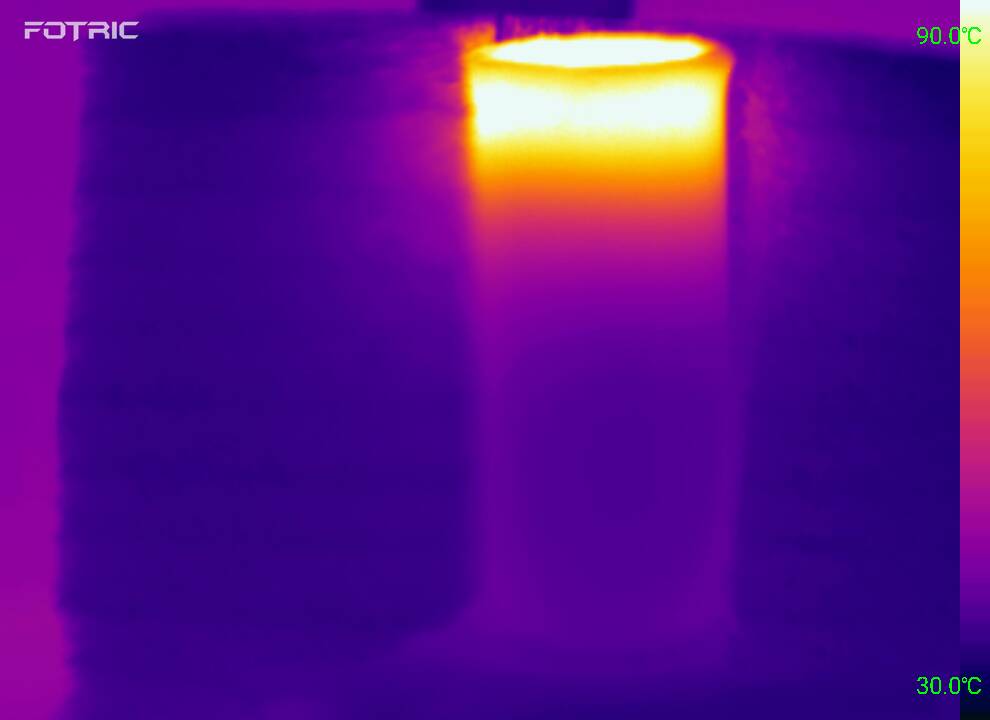

Supplement: Supplementary file 6 — Source Data [file 41467_2023_39190_MOESM6_ESM.zip › 1-original data/fig.6/fig.6e-up/1045.jpg]

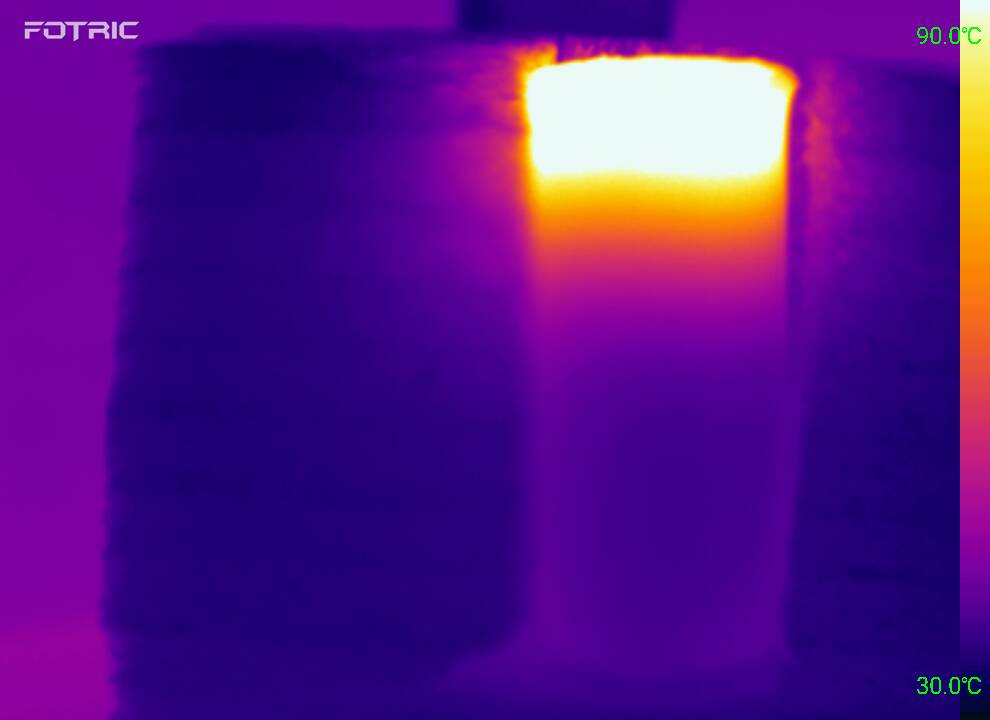

Supplement: Supplementary file 6 — Source Data [file 41467_2023_39190_MOESM6_ESM.zip › 1-original data/fig.6/fig.6e-up/1105.jpg]

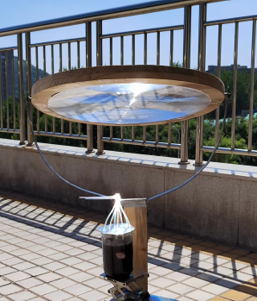

Supplement: Supplementary file 6 — Source Data [file 41467_2023_39190_MOESM6_ESM.zip › 1-original data/fig.7/Fig.7a.png]

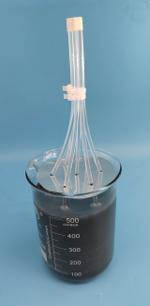

Supplement: Supplementary file 6 — Source Data [file 41467_2023_39190_MOESM6_ESM.zip › 1-original data/fig.7/Fig.7b.png]

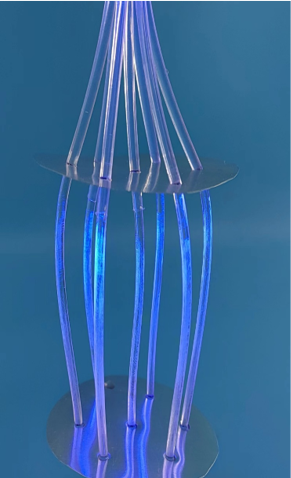

Supplement: Supplementary file 6 — Source Data [file 41467_2023_39190_MOESM6_ESM.zip › 1-original data/fig.7/Fig.7c.png]

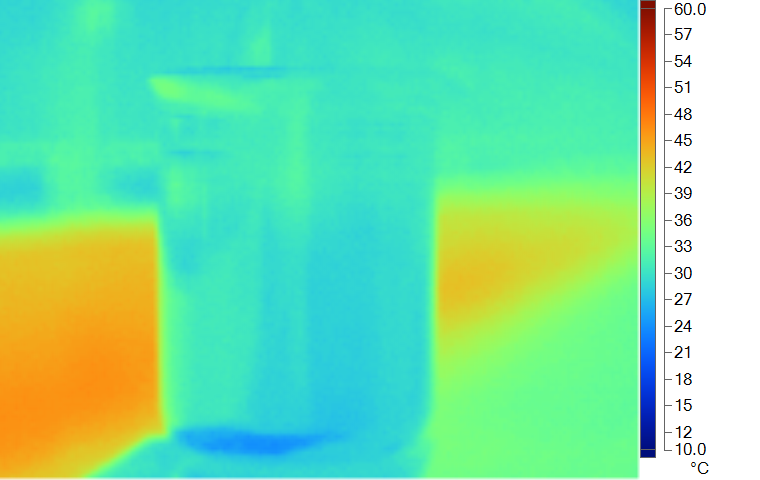

Supplement: Supplementary file 6 — Source Data [file 41467_2023_39190_MOESM6_ESM.zip › 1-original data/fig.7/Fig.7d.png]

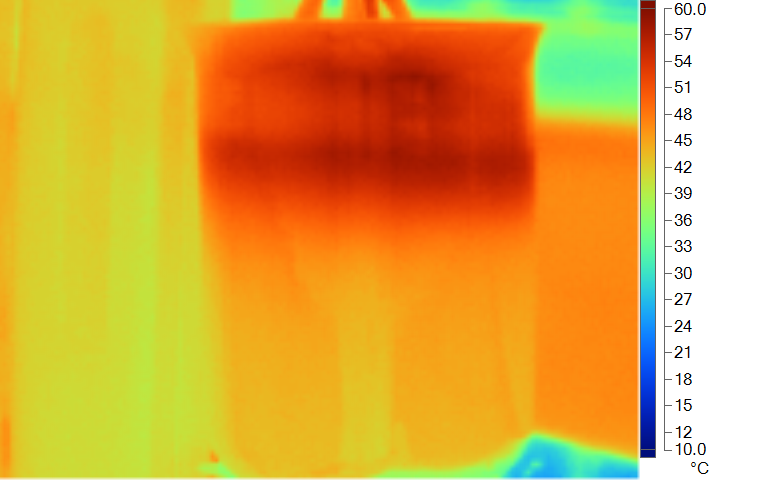

Supplement: Supplementary file 6 — Source Data [file 41467_2023_39190_MOESM6_ESM.zip › 1-original data/fig.7/Fig.7e.png]
